# Novel Immune Checkpoints in Esophageal Cancer: From Biomarkers to Therapeutic Targets

**DOI:** 10.3389/fimmu.2022.864202

**Published:** 2022-05-20

**Authors:** Xueyin Zhou, Ting Ren, Hongyuan Zan, Chunyan Hua, Xufeng Guo

**Affiliations:** ^1^School of Medicine, Wenzhou Medical University, Wenzhou, China; ^2^Department of Thoracic Surgery, Shanghai Chest Hospital, Shanghai Jiao Tong University, Shanghai, China; ^3^School of Basic Medical Sciences, Wenzhou Medical University, Wenzhou, China

**Keywords:** LAG-3, TIM-3, TIGIT, esophageal cancer, immunotherapy, biomarker

## Abstract

Esophageal cancer ranks as the sixth most common cause of cancer death worldwide. Due to the limited efficacy of conventional therapeutic strategies, including surgery, chemotherapy, and radiotherapy, treatments are still far from satisfactory in terms of survival, prompting the search for novel treatment methods. Immune checkpoints play crucial roles in immune evasion mediated by tumor cells, and successful clinical outcomes have been achieved *via* blocking these pathways. However, only a small fraction of patients can benefit from current immune checkpoint inhibitors targeting programmed cell death ligand-1 (PD-L1) and cytotoxic T-lymphocyte-associated protein-4. Unfortunately, some patients show primary and/or acquired resistance to immune checkpoint inhibitors. Until now, novel immune checkpoint pathways have rarely been studied in esophageal cancer, and there is a great need for biomarkers to predict who will benefit from existing strategies. Herein, we primarily discuss the roles of new immune checkpoints as predictive biomarkers and therapeutic targets for esophageal cancer. In addition, we summarize the ongoing clinical trials and provide future research directions targeting these pathways.

## 1 Introduction

Esophageal cancer (EC) is one of the most common cancers worldwide, ranking seventh in incidence and currently the sixth leading cause of cancer-related deaths (1). Nearly half of new cases worldwide are diagnosed in China every year, where the histological type is mainly esophageal squamous cell carcinoma (ESCC) (2). Various treatment strategies have been developed and implemented in the clinic, including surgery, radiotherapy, chemotherapy, and targeted gene therapy. In recent decades, with the promotion of multidisciplinary diagnosis and treatment, the overall survival (OS) of EC has been greatly improved, but the results are still unsatisfactory. epidermal growth factor receptor- (EGFR-), human epidermal growth factor receptor 2 (HER2), vascular endothelial growth factor receptor- (VEGFR-), and cellular-mesenchymal epithelial transition factor- (c-MET-) targeted therapy have been well studied and showed encouraging efficacy. However, the low therapeutic efficacy of targeted therapy limits its application during the treatment of EC. Therefore, there is an urgent need to exploit alternative therapeutic strategies with novel mechanisms of action that ameliorate antitumor efficacy and overcome adverse effects, thus improving the prognosis of patients (3, 4).

Progressive insight into tumor immunology and immunosuppressive environment that favors tumor growth has paved the way for the advent of immunotherapy based on immune checkpoints (ICs) targeting programmed cell death protein 1 (PD-1) and cytotoxic T-lymphocyte-associated protein-4 (CTLA-4), which have revolutionized current therapeutic methods. The safety and efficacy of immune checkpoint inhibitors (ICIs) have been verified in several clinical trials that indicated that IC blockade is a promising method in first- and second-line treatment for advanced EC (5, 6). However, only a small proportion of patients showed long and lasting responses (7). In addition, some patients receiving anti-CTLA-4 monoclonal Ab (mAb) and anti-PD-1 mAb have shown resistance to ICIs to varying degrees due to tumor-extrinsic and/or -intrinsic factors (8–10). Furthermore, immune-related adverse events (irAEs) including colitis and hepatitis have led to the discontinuation of treatment (11). Therefore, it is essential to find an alternative IC pathway with long-term efficacy, extensive beneficiaries, and controllable toxicity in order to offer more feasible options for those who do not benefit from ICIs targeting PD-1 and CTLA-4.

**Figure 1:**
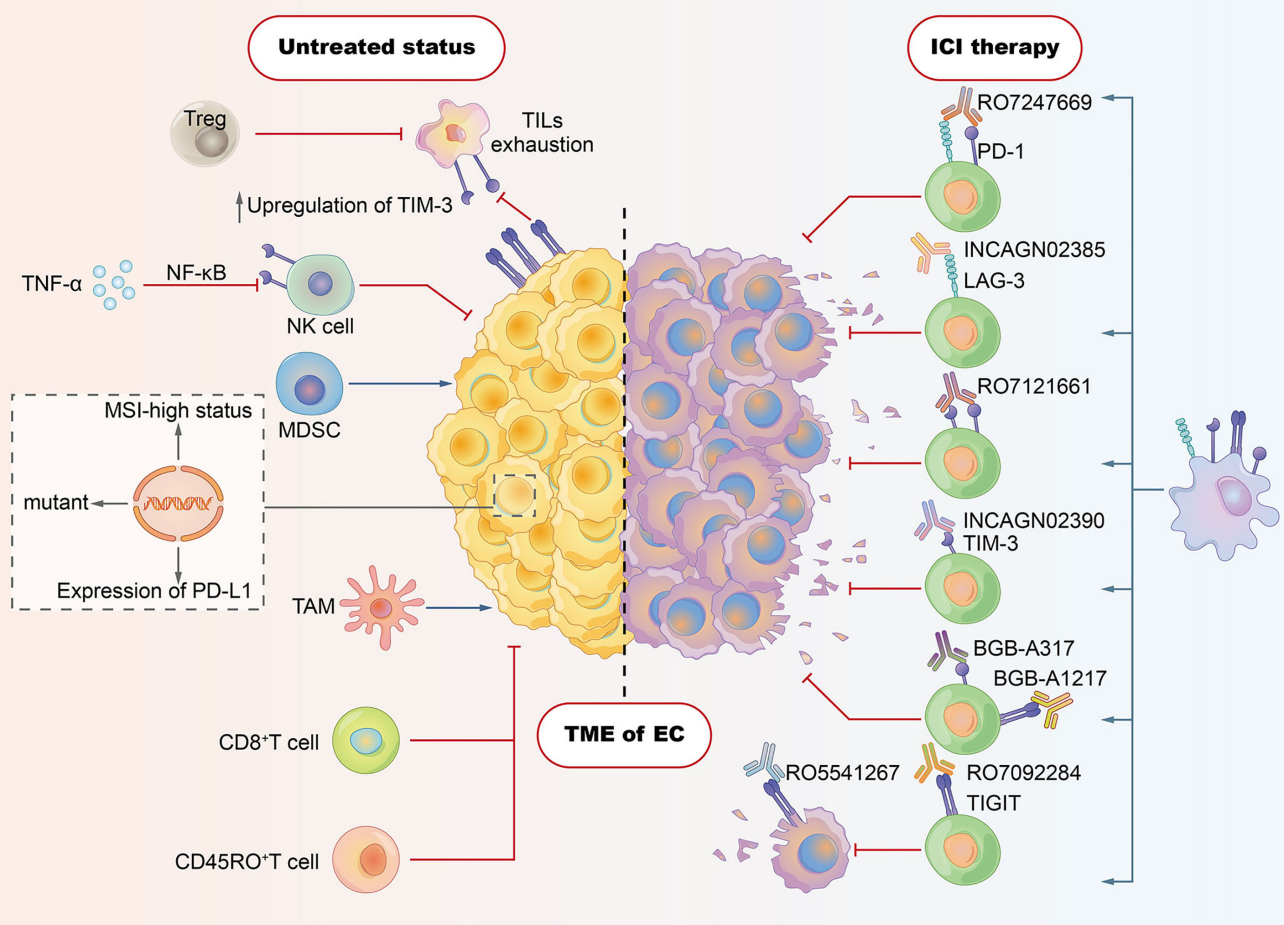
Tumor microenvironment (TME) in esophageal cancer: tumor cells evade host immunity *via* a series of cell-extrinsic factors that comprise the TME. Immune checkpoint inhibitors (ICIs) are designed to reverse the TME of immunosuppressive effects, thereby enhancing patients’ immune responses against tumors. As shown in the left section, CD8^+^ T cell, CD45RO^+^ T cell, and NK cell contribute to the antitumor immune response. However, some subsets of cells with negative immunomodulatory function play crucial roles in suppressing antitumor effects. Regulatory T cells (Tregs) have been shown to dampen the activity of tumor-infiltrating lymphocytes (TILs). Tumor-associated macrophages (TAMs) and myeloid-derived suppressor cells (MDSCs) also boost the immune evasion of tumor cells. Some tumor cell–intrinsic factors (e.g., PD-L1 expression, tumor mutation load, and MSI-high status) account for the cancer resistance to ICIs. The expression of ICI such as TIGIT and PD-1 may be associated with the exhaustion of TILs. Induced frequency of NK cells in EC is ascribed to TNF-α, which is able to induce the expression of TIM-3 *via* the NF-κB signaling pathway. The right section depicts the novel ICIs strategies targeting LAG-3, TIM-3, and TIGIT, which aim to reverse the exhaustion or dysfunction states of T cells.

Lymphocyte activation gene-3 (LAG-3), T-cell immunoglobulin (Ig) and mucin domain-containing protein-3 (TIM-3), and T-cell Ig and ITIM domain (TIGIT) are candidates for the next generation of ICs. Preclinical data have shown their notable immune inhibitory effects toward lymphocytes, which indicates that the blockade of these ICs could normalize immunity in the tumor microenvironment (TME) and exert robust antitumor effects (12–14) (**Figure 1**). For the sake of its synergetic immunosuppressive effects with PD-1, the dual blockade of new ICs with PD-1 has shown encouraging results in some preclinical trials of some types of cancer, which also brings hope to immunotherapy for EC (15–17).

Although the road ahead is promising, the biology of the new targets is incompletely understood, especially in EC. Obtaining deeper insight into their biology is conducive to optimize clinical design with optimal efficacy and fewer toxicity. In the scope of this review, we comprehensively summarize the characteristics of these novel ICs and discuss the immune escape pathways mediated by them. To date, the downstream signaling pathway of LAG-3 remains obscure, although the latent downstream signal pathway of LAG-3 has been described in different studies and will be described in this review. Furthermore, we highlight the potential use of these ICs as diagnostic and prognostic biomarkers as well as therapeutic targets, which could provide meaningful research directions for EC.

## 2 Novel Immune Checkpoints

### 2.1 Lymphocyte Activation Gene 3

#### 2.1.1 Structure and Expression

LAG-3 (lymphocyte activation gene 3, CD223) is a new type I acid transmembrane protein consisting of 498 amino acids belonging to the immunoglobulin superfamily. The LAG-3 gene is mapped on chromosome 12, which is located adjacent to the CD4 gene. Although there are similarities in chromosome location, approximately 20% of amino acid sequences are identical (18, 19). Like CD4, the extracellular region of LAG-3 is composed of four immunoglobulin superfamily domains (D1–D4) (8, 20). Unlike CD4, LAG-3 uses an “extra loop” consisting of 30 amino acids in the D1 domain that bind to MHC class II molecules with greater affinity than CD4. The D2 domain is important for the LAG-3/MHC class II binding and participating in the positioning of the D1 domain (21). In contrast to this conclusion, a rat mAb (clone C9B7W) to mouse LAG-3 that binds to the D2 domain could not interfere with LAG-3 binding to MHC class II (7). A longer connecting peptide (CP) is located between D4 and the transmembrane region, causing LAG-3 to be cleaved by two transmembrane metalloproteases (ADAM10 and ADAM17) in the CP, thereby generating a soluble form of LAG-3 (sLAG-3) (8, 22, 23). The cytoplasmic region of LAG-3 incorporates three motifs (20). The first is the FxxL motif, which includes two putative serine phosphorylation sites in humans (14, 24). This leads to the suspicion that the FxxL motif is crucial in signal transmission because serine phosphorylation can activate the protein. However, the serine mutation does not impact on the activity of LAG-3. In addition, there are no tyrosine or threonine residues, limiting phosphorylation events (25). The second is called the “KIEELE” motif. Workman et al. (26) demonstrated that the “KIEELE” motif is required for the inhibitory effects of LAG-3 on T cells, while effects on downstream signaling and function are still unclear. In fact, another study found that the KIEElE motif had no influence on LAG-3 function after deletion (25). The third is the EP motif composed of proline dipeptide and repetitive glutamate (27, 28). It has been demonstrated that the key to counteract CD3/TCR activation is binding between the EP motif and the LAG-3-associated protein (LAP). However, LAG-3 mutants lacking the EC motif still remain active, suggesting that this motif is not indispensable for LAG-3 (27, 28).

Due to the special intracellular structure, the signaling pathway pattern of LAG-3 remains obscure. Several studies have confirmed that LAG-3 is an inhibitory receptor for T lymphocyte activation and its presence can inhibit IL-2 production by CD4^+^ T cells (29). LAG-3 exerts an inhibitory effect on the activation of the CD3/TCR pathway and inhibits CD3-induced Ca2^+^ production (28). According to previous studies, the activation of the CD3/TCR pathway induces an efflux of Ca^2+^ from the endoplasmic reticulum (ER), which binds to the calcium sensor protein calmodulin. The calcium sensor protein calmodulin, in turn, activates calcineurin (CaN). CaN dephosphorylates and activates the NFAT transcription factor, which translocates to the nucleus to promote gene expression in cooperation with multiple transcription molecules to produce IL-2 (30, 31). Based on the above evidence, it can be speculated that the CaN/NFAT signaling pathway is most likely the downstream signaling pathway through which LAG-3 inhibits CD3/TCR function.

**Figure 2:**
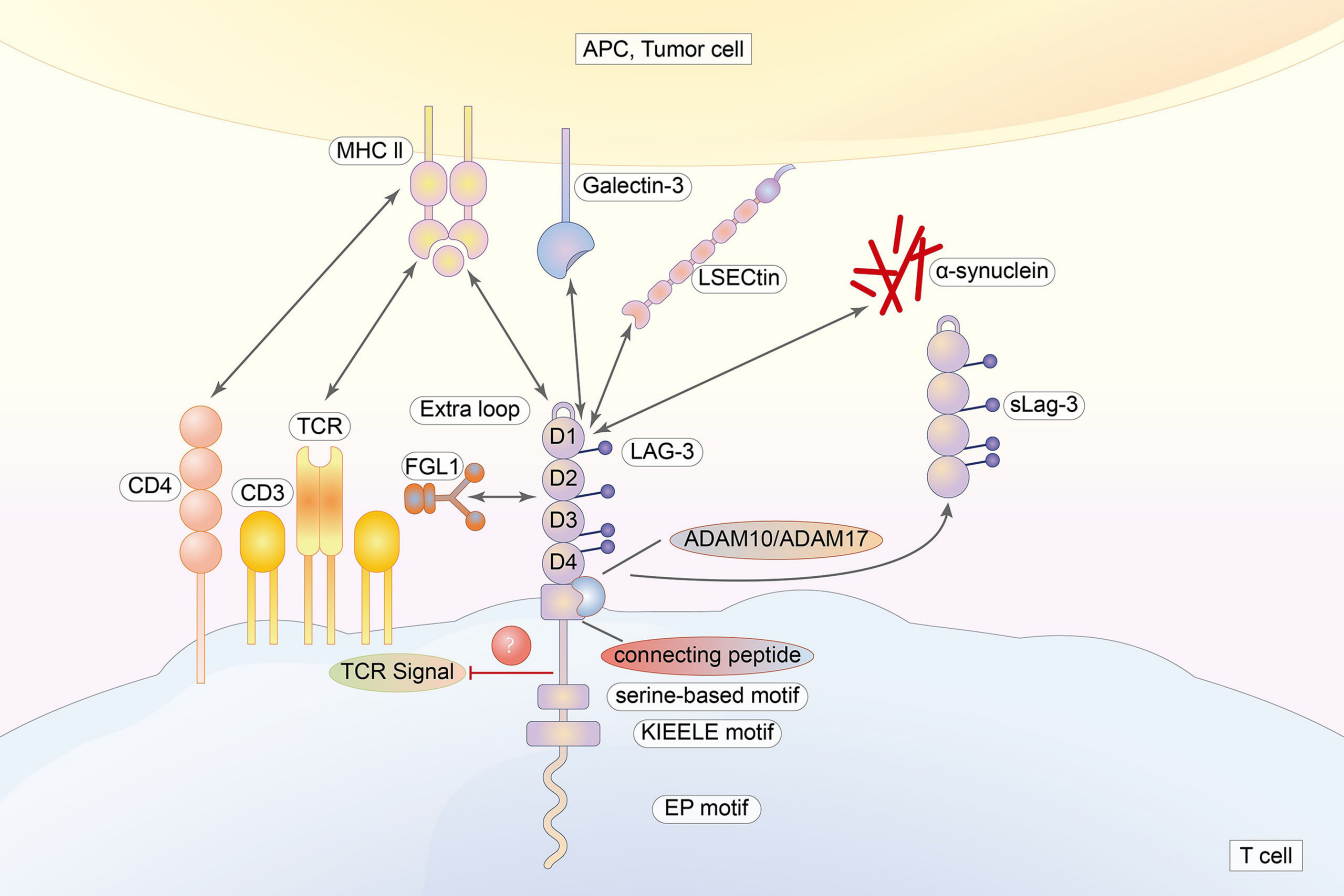
The extracellular region of LAG-3 is composed of four immunoglobulin superfamily domains (D1–D4). The cytoplasmic tail of LAG-3, incorporating three regions, is highly conversed. LAG-3 binds to MHC class II with higher affinity than that of CD4 *via* an “extra loop” in the D1 domain. Galectin-3, LSECtin, FGL, and α-synuclein are also ligands for LAG-3.

LAG-3 expression was found mainly in conventional T cells (32), regulatory T cells (Tregs), and unconventional T cells, including mucosal-associated invariant T (MAIT) cells, γδT cells, natural killer T (NKT) cells, and invariant NKT (iNKT) cells (21); NK cells (33); plasmacytoid dendritic cells (pDCs) (33); neurons (34); and tumor-associated macrophages (TAM) (21). Certain conditions, such as continued antigen stimulation and exposure to cytokines (including IL-2, IL-7, and/or IL-12 and IFN-γ) induce the expression of LAG-3 on T cells and NKT cells (35). NKG2C^+^ NK cells, acting as a subpopulation of NK cells, express LAG-3 in response to the IL-15 and NKG2C agonist (35). Within the TME, increased expression of LAG-3 with other ICs such as TIM-3, TIGIT contributes to T-cell dysfunction (18). Wang et al. (36) demonstrated that the expression level of LAG-3 is higher in ESCC tissues than in normal tissues. This may signify that the blockade of LAG-3 might exert antitumor effects in the immunotherapy of EC patients with a positive expression of LAG-3 (**Figure 2**).

#### 2.1.2 Ligands and Axis

Given the structural similarity between LAG-3 and CD4, major histocompatibility complex class II (MHC II) molecules expressed on antigen-presenting cells (APCs) and tumor cells are the canonical ligands for LAG-3 (27, 37–39). LAG-3 plays vital roles in the downregulation of the proliferation (40), activation, and homeostasis of T cells through binding to MHC II, while the exact mechanism of signal transmission remains unclear (27, 36). LAG-3 affects the function of CD8^+^ T cells that do not express MHC II molecules (21). Additionally, some monoclonal antibodies enhance T-cell functions that do not interfere with the binding between LAG-3 and MHC II. These data have led to the doubt that there may be alternative ligands for LAG-3 (41).

Currently, four molecules have been identified as ligands for LAG-3: galectin-3, liver sinusoidal endothelial cell lectin (LSECtin), fibrinogen-like protein 1 (FGL1), and α-synuclein (7, 21, 42). Galectin-3 expression is not limited to tumors. Epithelial cells and immune-related cells such as DCs, monocytes, and macrophages were also shown to express LAG-3 (43). In most tumors of the digestive tract and the bloodstream of cancer patients, the expression level of galectin-3 is increased (42, 44). LAG-3 was also suggested to be indispensable for suppressing the secretion of IFN-γ produced by T cells in a galectin-3-dependent manner *in vitro* (45). LSECtin expression was found on DC (46, 47). Furthermore, fibrinogen-like protein 1 (FGL1) is regarded a major ligand of LAG-3 that is secreted by the liver and upregulated in several types of cancers (48). FGL1 does not appear to compete with MHC II in binding to LAG-3, suggesting that MHC II and FGL1 may have different active binding sites (49). Further, it seems possible that LAG-3 blockade cannot completely block its inhibitory effects. LAG-3 also binds to α-synuclein, which is an increased risk of Parkinson’s disease (50) (**Figure 2**).

### 2.2 TIM-3

#### 2.2.1 Structure and Expression

TIM-3 or T-cell immunoglobulin and mucin-domain containing-3 is a type I transmembrane protein, which consists of 302 amino acids and belongs to the immunoglobulin superfamily (51). The TIM-3 gene, HAVCR2, is located in 5q33.2 of the human genome, which is related to asthma, allergies, and autoimmunity. It has an extracellular domain composed of an N-terminal immunoglobulin domain at the far end and a mucin domain containing latent sites for O-linked sugars at the near end. Between the mucin and transmembrane domains, the stalk domain includes sites for N-linked glycosylation. The transmembrane domain is followed by a cytoplasmic tail.

**Figure 3:**
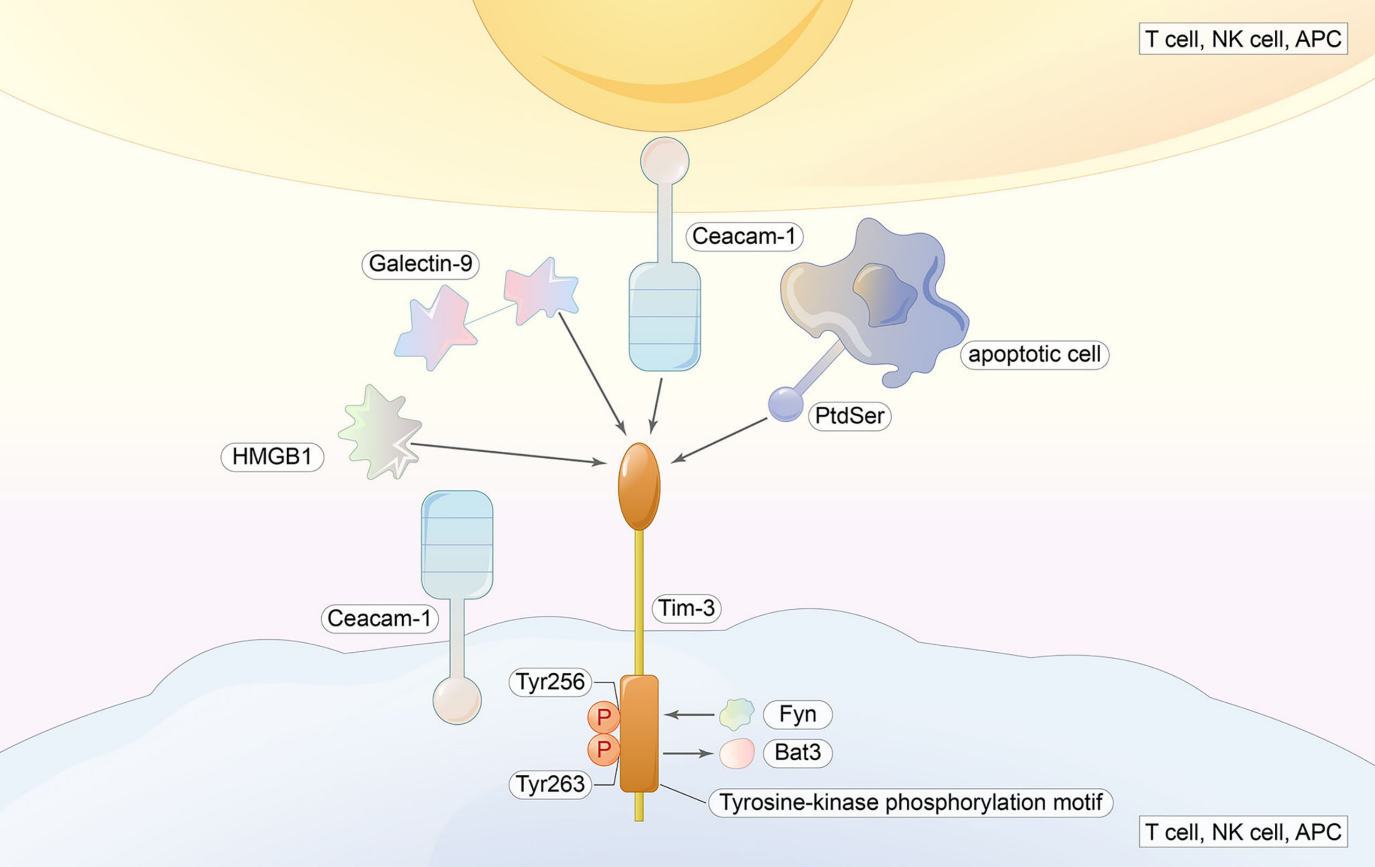
TIM-3 ligands include galectin-9, HMGB1, Ceacam-1, and PtdSer. Ligand binding induces the phosphorylation of Tyr256 and Tyr263 as well as the release of Bat3 from the tail, and Fyn will bind to the same region; thus, the inhibitory function is initiated.

Many types of murine and human immune cells express TIM-3 (52, 53), which is an inhibitory receptor for CD4^+^ and CD8^+^ T cells that yield IFN-γ. The co-expression of PD-1 and TIM-3 a characteristic of serious T-cell failure, largely due to considerably reduced cytokine production and the inhibition of T-cell proliferation (54). Early studies of TIM-3 have shown that its inhibitory effect causes the -inhibition of effector Th1 responses in models of multiple sclerosis and autoimmune disease in mice with type 1 diabetes. The generation of IFN-γ and TNF from reactive T cells is increased and the development of peripheral tolerance is inhibited by blocking anti-TIM-3 signaling (53, 55) and generating soluble forms of TIM-3, TIM-3.FC (56). In subsequent human studies, increased IFN-γ production on CSF-produced T-cell clones from patients with multiple sclerosis (MS) was associated with a decreased expression of TIM-3, indicating that impaired T-cell tolerance is associated with the dysregulation of TIM-3 expression. The following studies have shown that non-T cells such as NK cells, dendritic cells (DC), and macrophages also express TIM-3 (51, 57). Another study found that the inhibitory function of TIM-3 working in the non-T-cell population was consistent with the characterization of T cells (**Figure 3**) (51).

#### 2.2.2 Ligands and Axis

TIM-3 has several ligands (Gal-9, PtdSer, HMGB1, and CEACAM-1). The interaction of TIM-3 with Gal-9 mediates effector T-cell apoptosis through Calc-calprotease caspase 1, which also increases IFN-γ production in NK. However, a study in chronic hepatitis B found the opposite (58).

The importance of exploring the downstream signaling pathway of TIM-3 is highlighted by the fact that it is an important regulator of effector T cells. No classical signaling motif is contained in the cytoplasmic tail of TIM-3, which is similar to LAG-3 (59). Instead, mouse and human TIM-3 both have five conserved tyrosine residues in the cytoplasmic tails where Y256 and Y263 can be phosphorylated by Src kinases (60) or ITK (61). Y256 and Y263 are two sites involved in the binding of Bat3 (HLA-B-associated transcript 3), p85 PI3K, Fyn, and Lck to the C-terminal tail of TIM-3 (60, 62). When ligand-mediated TIM-3 signaling is absent, Bat3 binds to it and blocks SH2 domain-binding sites in the tail. Subsequently, Bat3 recruits the catalytically active form of Lck, forming an intracellular complex that preserves and possibly promotes T-cell signaling (62). In contrast, T cells deficient in Bat3 exhibit an increase in pY505 Lck, which is the catalytically inactive form of Lck (62). The binding of Galectin-9 and Ceacam-1 to TIM-3 induces the phosphorylation of Y256 and Y263, as well as the release of Bat3 from the tail. Consequently, TIM-3-mediated T-cell inhibition is promoted by the permissive binding of SH2-containing Src kinases and is thus subsequent to the regulation of TCR signaling (62, 63). In addition, Fyn binds to the same region as Bat3. Fyn is involved in T-cell anergy (64) and is a key kinase that activates a phosphoprotein associated with glycosphingolipid microdomains (PAG), which recruits Csk to suppress Lck function (65, 66). As Fyn and Bat3 bind to the same domain in TIM-3, it is likely that a change between TIM-3-Fyn and TIM-3-Bat3 may cause the changes in TIM-3 function from that in allowing T-cell receptor (TCR) signaling to the inhibiting upstream TCR signaling. Consistent with the data from some trials, the loss of Bat3 leads to the dephosphorylation and degradation of TCRζ (62) (**Figure 3**).

### 2.3 T-Cell Immunoglobulin and Immunoreceptor Tyrosine-Based Inhibitory Motif Domain

#### 2.3.1 Structure and Expression

**Figure 4:**
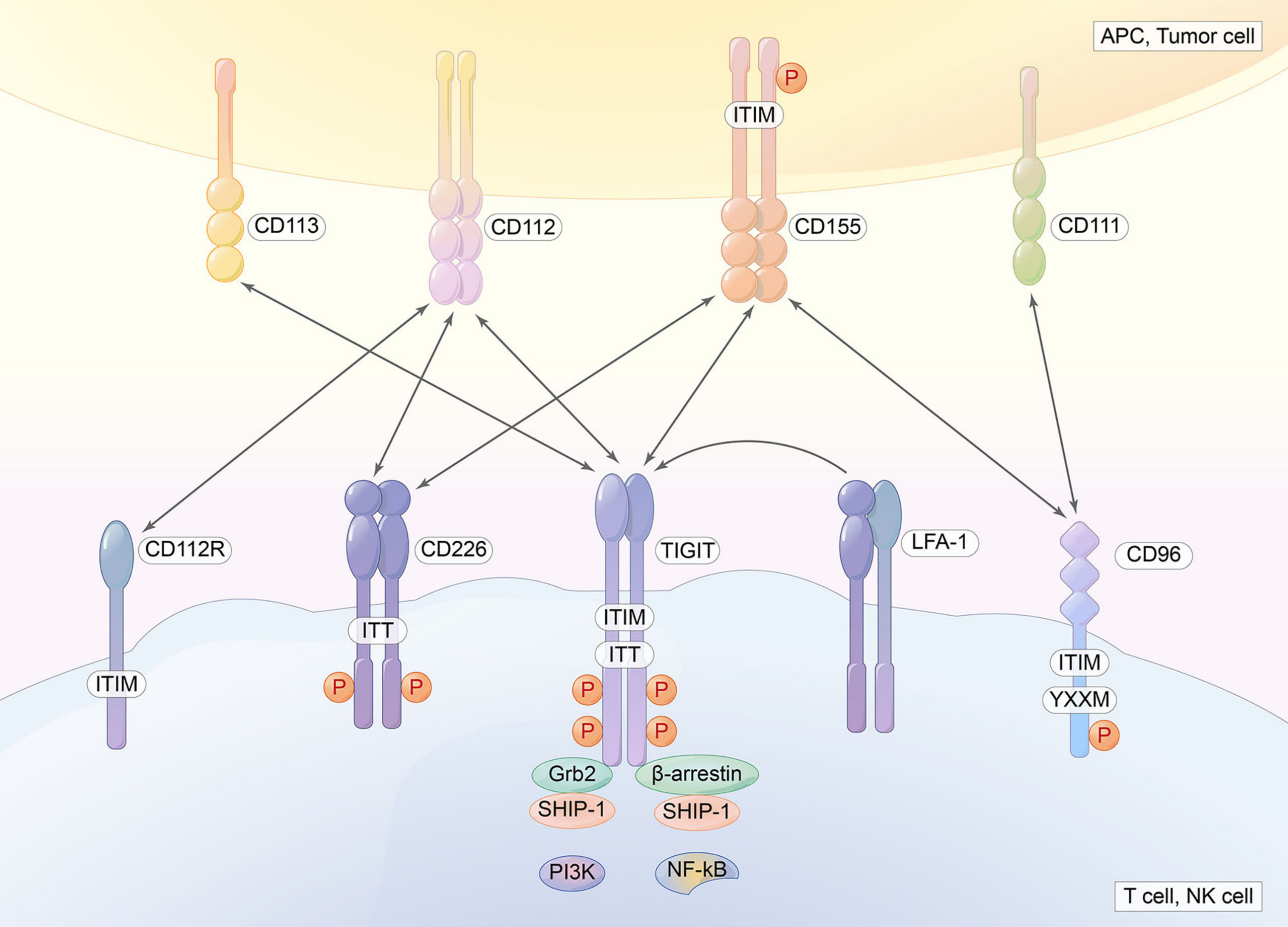
TIGIT contains an extracellular immunoglobulin variable domain, a type I transmembrane domain, and a short intracellular domain with one immunoreceptor tyrosine-based inhibitory motif (ITIM) and one immunoglobulin tyrosine tail (ITT) motif. CD155, CD112, and CD113 are ligands for TIGIT.

TIGIT, also called the T-cell immunoglobulin and immunoreceptor tyrosine-based inhibitory motif domain, is a novel inhibitory IC receptor (67). The TIGIT gene is located on human chromosome 3q13.31 and encodes a 244 amino acid transmembrane glycoprotein. It has an extracellular immunoglobulin variable domain: a type I transmembrane domain and a short intracellular domain with an immunoreceptor tyrosine-based inhibitory motif (ITIM) and an immunoglobulin tyrosine tail motif (ITT). TIGIT is generally expressed in T cells and NK cells, including CD4^+^ T cells, CD8^+^ T cells, and Tregs. High expression of TIGIT is associated with the exhaustion of tumor-infiltrating NK cells. Therefore, the deficiency of TIGIT alleviates NK-cell exhaustion and slows tumor growth, which elicits potent antitumor immunity (68). Tregs expressing TIGIT are a distinct subset, which can particularly suppress Th1 and Th17 pro-inflammatory responses and promote the suppression of effector T-cell proliferation. We also found that CD8^+^TIGIT^+^ cells display a dysfunctional subset with less IL-2 and TNF-α production, however, along with high IL-10 secretion. Furthermore, TIGIT^+^ Tregs upregulate TIM-3 within the TME. They suppress antitumor immunity by controlling cytotoxic T-cell activity (69). By binding to poliovirus receptors and modulating dendritic cell cytokine production, TIGIT plays a vital role in immunosuppressive effects (70) (**Figure 4**).

#### 2.3.2 Ligands and Axis

TIGIT has many ligands, CD155 (PVR or Necl-5), CD112 (nectin-2, also known as PRR2 or PVRL2), and CD113, CD155, CD112, CD113, and CD111, which are expressed in APCs or tumor cells. TIGIT, CD112R, and CD155 deliver inhibitory signals to cells. However, DNAM-1 delivers activating signals because it contains an immunoglobulin tyrosine tail (ITT)-like domain. CD96 in humans and mice contains an ITIM domain, but human CD96 also contains an YXXM motif. CD96 can inhibit mouse cells and NK cells. TIGIT binds CD155 with the highest affinity, followed by CD96 and then DNAM-1, CD112R, and Nectin4 (a ligand for TIGIT, which binds only to TIGIT). Competing with other counterparts (CD266 and CD96), TIGIT exerts immunosuppressive effects (8, 71, 72). PVRIG is a coinhibitory receptor that binds to PVRL2 (73). CD155 is always overexpressed in human malignant tumors. The overexpression of CD155 promotes the invasion and migration of tumor cells and is associated with a poor prognosis in many types of tumors. The balance between CD155/CD226 and CD155/TIGIT or CD155/CD96 maintains normal NK- and T-cell function (73). Weulersse et al. (74) revealed that the absence of CD226 expression identifies hyporeactive human CD8^+^T cells. IL-15, which triggers NK-cell proliferation and potentiates NK-cell function, induces the upregulation of the expression of both CD226 and TIGIT (75). CD226 is associated with the integrin LFA-1 and delivers a positive signal (**Figure 4**).

### 2.4 Other Newly Emerging Immune Checkpoints

Despite TIM-3, LAG-3, and TIGIT, other newly emerging ICs, for example, V-domain Ig suppressor of T cell activation (VISTA), B7-H3, B and T lymphocyte attenuator (BTLA), and Siglec-15, have been identified to date and are in various stages of preclinical and clinical development in combination with IC therapy. They are promising methods to improve response rates in EC (76–79).

VISTA is a type I transmembrane protein with high-level conversation and limited homology to other members of the B7 family (80). VISTA negatively regulates the activation of T cells and induces the expression of Foxp3. VSIG-3 is a VISTA ligand whose interaction with VISTA was shown to inhibit T-cell proliferation and cytokine production. Anti-VISTA neutralization antibodies were shown to mitigate the inhibition of T cells (81). The expression of VISTA was found in tumor cells, CD68 ^+^ TILs, and CD4^+^ TILs in tissues of esophageal adenocarcinoma (82). In contrast to the previous concept that VISTA is an IC with a negative immune regulator, VISTA might serve as a co-stimulatory molecule in esophageal adenocarcinoma (76). Patients with VISTA-positive expression achieved a higher median OS compared to patients with VISTA-negative expression. The subsequent subgroup analysis revealed that tumors with VISTA-positive TILs exhibited higher OS in pT1/2 tumors compared to patients with no VISTA expression on TILs (83) (**Table 1**).

**Table 1:** The therapeutic and prognosis value of novel immune checkpoint pathways in EC.

Immune checkpoint	Species	Site of expression	Role	Regulation mechanism	Ref.
**LAG-3**	Human	EAC tissues	Reduced recurrence rate Improved OS (pT3/4)	Patients with LAG-3 expression are correlated with improved OS compared to those with negative LAG-3 expression.	(84)
	Human	ESCC tissues	Controversial	High LAG-3 expression is correlated with longer OS and PFS, while LAG-3^+^/CTLA-4^+^CD8^-^ patients showed unfavorable RFS and OS.	(36, 85)
**TIM-3**	Human	ESCC tissues	Suppressed anti-tumor immunity	Patients with TIM-3^+^PD-1^+^CD8 low had the worst RFS and OS. Patients with TIM-3^-^PD-1^-^CD8^high^ had the best RFS and OS.	(86)
	Human	ESCC tissues	Induced frequency of NK cells	TNF-α was able to induce the expression of TIM-3 on NK cells through the NF-κB signaling pathway.	(86)
**TIGIT**	Human	TILs	TIL exhaustion	TIGIT was upregulated in TILs and might be associated with TIL exhaustion.	(87)
	Human	TILs	Unfavorable prognosis	PD-1^+^/TIGIT^+^ TILs had poor prognosis in primary ESCC.	(88)
	Human	EC cells	Unfavorable OS and PFS	Highly expressed TIGIT was associated with unfavorable OS and PFS.	(89)
**VISTA**	Human	EAC	Higher median OS	Patients with VISTA-positive expression are correlated with high median OS compared with patients with VISTA-negative expression.	(83)
	Human	TILs	Higher OS in pT1/2	Tumors with VISTA-positive TILs exhibited higher OS in pT1/2 tumors compared to patients with no VISTA expression on TILs.	(83)
**B7-H3**	Human	ESCC tissues	Tumor invasion and suppressed anti-tumor immunity mediated by T cells	High B7-H3 expression was correlated with tumor invasion and suppressed anti-tumor immunity mediated by T cells.	(90)
	Human	ESCC tissues	Enhanced intensity of Foxp3^+^ T cells and infiltration of TAMs	The expression of B7-H3/B7-H4 was positively associated with the intensity of Foxp3^+^ T cells and the presence of TAMs.	(91)
**BTLA**	Human	ESCC tissues	Lower OS	The positive expression of BTLA is associated with worse OS rates compared to the BTLA-negative expression group.	(92)
	Human	ESCC tissues	Lower OS	The co-expression of PD-1 and BTLA or TIM-3 and BTLA was correlated with worse OS.	(92)

ESCC, esophageal squamous cell carcinoma; EAC, esophageal adenocarcinoma; TILs, tumor-infiltrating lymphocytes; OS, overall survival; PFS, progression-free survival; RFS, recurrence-free survival; TAMs, tumor-associated macrophages.

B7-H3 is a type I transmembrane protein that belongs to the B7 family (93). Initial studies reported that B7-H3 is a co-stimulatory molecule, while recent studies have demonstrated that it was an inhibitor of T cells. The ligand for B7-H3 has not been defined, which may partly be accounted for its versatile functions from other studies (93). The expression of B7-H3 is significantly higher in EC cells, while no or a weak expression of B7-H3 has been found in normal esophageal tissues (90). This result is in line with previous studies that identified B7-H3 mRNA in a wide range of normal human tissues, while the B7-H3 protein is expressed at low levels (77). *In vivo* and *in vitro* studies have revealed that high expression of B7-H3 was correlated with tumor invasion and suppressed antitumor immunity mediated by T cells displayed by enhanced intensity of Foxp3^+^ T cells and the infiltration of TAM. Furthermore, the high expression of both B7-H3 and B7-H4 has been associated with increased invasion and a high TNM stage (90, 91) (**Table 1**).

BTLA is a type I glycosylated transmembrane protein that belongs to the CD28 superfamily (94). BTLA is expressed on lymphocytes in ESCC (92). The herpesvirus entry mediator (HVEM) is considered a ligand for BTLA whose interactions lead to the inhibition of T-cell division. BTLA competes with LIGHT and lymphotoxin-α for binding to HVEM (80). BTLA has been proposed as an independent factor for the evaluation of OS in patients with ESCC. The positive expression of BTLA is associated with worse OS rates compared to the negative expression group of BTLA. The co-expression of PD-1 and BTLA or TIM-3 and BTLA was also correlated with worse OS (92) (**Table 1**).

Siglec-15 is a sialic acid-binding immunoglobulin-like lectin belonging to the Siglec gene family member (95). Siglec-15 mRNA is not detected in most normal human tissues and immune cells except for macrophages. However, Siglec-15 could be detected in TAMs and human cancer cells. IFN-γ, an inducer of PD-L1 expression, has also been shown to suppress Siglec-15 expression, which may partly explain that the pattern of Siglec-15 expression is mutually exclusive to that of PD-L1 (96, 97). This interesting finding indicated that blocking Siglec-15 could be effective for patients who do not benefit from anti-PD-L1 therapy and the proliferation of low PD-L1 expression. A significant finding is that Siglec-15 knockout mice did not develop autoimmune-like diseases and displayed reduced tumor growth with NK-cell and CD8^+^ T-cell infiltration. In addition, enhanced cytokine production has also been detected, which was in line with the concept of the normalization of cancer immunotherapy previously proposed (79, 98).

## 3 Preclinical Data in Esophageal Cancer

There has been no consensus on the exact role of sLAG-3. The cleavage of LAG-3 is mediated by ADAM10 and ADAM17 for efficient T-cell proliferation and cytokine production (23). Therefore, sLAG-3 is likely “wasted” in terms of regulating T-cell function (99). Conversely, the serum levels of sLAG-3 in patients diagnosed with advanced EC were higher than in healthy controls, indicating the crucial role of sLAG-3 (100). sLAG-3 was demonstrated to prevent monocyte differentiation from macrophages and DC (101). CD4^+^ T-cell clones release soluble LAG-3-related peptides after activation, which is positively associated with the production of IFN-γ (102). In addition, sLAG-3 may function as an immune adjuvant. In mouse models, sLAG-3 was capable of enhancing antitumor effects mediated by T cells in response to the irradiated tumor cell vaccine (103). It is plausible that sLAG-3 binding to its ligand inhibits its inhibitory effects. However, there is no ample evidence to support the conjecture (99). LAG-3V3, which contains 3 domains (D1-D3), another soluble form of LAG-3, has been proposed as a serological marker of Th1 activity (104).

Although the past decades have witnessed the improvement of therapeutic methods, the prognosis of EC is still far from satisfactory. Thus, there is an urgent need to find a viable biomarker that is able to predict the prognosis of EC patients (105). In fact, the evaluations of LAG-3 as a biomarker have been confirmed in several tumor types. In hepatocellular carcinoma (HCC), higher densities of LAG-3^+^cells were associated with shorter OS and disease-free survival (DFS) (106). Consistent with this result, LAG-3 expression was negatively correlated with OS in colorectal cancer (107). In patients diagnosed with locally advanced esophageal adenocarcinoma, the complete pathological response (CR), LAG-3 and CXCL9 were more predictive than CR alone in terms of DFS, which is correlated with the reduced rate of recurrence (108). Patients with higher LAG-3 expression have shown a correlation with better OS compared to those with negative LAG-3 expression. However, such a survival benefit is only detectable in pT34 tumors, while no difference was found in pT1/2 tumors (84). However, the role of LAG-3 used as a prognostic biomarker for ESCC remains controversial. Zhang et al. (85) demonstrated that high expression of LAG-3 is correlated with longer progression-free survival (PFS) and OS while the worst recurrence-free survival (RFS) and OS were found in patients with LAG-3^+^/CTLA-4^+^CD8^−^ cell populations (36). This contradictory pattern has also been observed in breast cancer. In ER^-^ and ER^+^ breast cancer, high expression of LAG-3 predicted a favorable outcome (109, 110). However, dual-positive LAG-3 and PD-1 were correlated with an unfavorable prognosis in terms of decreased DFS (111). In summary, LAG-3 may act as a promising biomarker for locally advanced esophageal adenocarcinoma. As for ESCC, three reasons may explain the paradox: First, different definitions of positive expression of LAG-3; second, different experimental parameters; third, as mentioned above, the expression of LAG-3 is induced by IFN-γ, which is a cytokine with antitumor effects. According to the results of Zhang et al. (85), the elevated level of IFN-γ may explain the variability of the landscape of LAG-3 expression. From a clinical perspective, sLAG-3 could also serve as a stage and diagnostic biomarker. For example, a high level of sLAG-3 has been correlated with an advanced tumor stage in patients with clear cell renal cell cancer (ccRCC) and a better prognosis in gastric cancer (112–115). Little is known about the diagnostic and prognostic values of sLAG-3 for EC, which is an area that needs to be studied.

Synergy and cooperative interactions between inhibitory pathways in cancer are pivotal regulators of immune escape in cancer. The synergy between LAG-3 and PD-1 was demonstrated to fortify tumor-induced tolerance. The dual blockade of these ICs showed more potent immune responses than monotherapy (16). A novel fully human antibody targeting LAG-3 named LBL-007 was found to block the interaction between LAG-3 and MHC II or LSECtin. The combination of anti-PD-1 antibody and LBL-007 has enhanced inhibitory effects on tumor growth than either monotherapy (116). Because it can block the interaction of LAG-3 between its two ligands at the same time, and its combination with anti-PD-1 antibody has a better curative effect than monotherapy, this may provide a new mechanism to alleviate the inhibitory effects against T cells.

TNF-α, which induces TIM-3 expression in T cells, and TGF-β, which induces TIM-3 expression in macrophages, are significant inflammatory factors in the TME (117, 118). Through the NF-κB pathway, TNF-α can induce the expression of TIM-3 in NK cells, the upregulation of TIM-3 may be correlated with NK-cell dysfunction in the EC microenvironment, and the frequency of peripheral TIM-3^+^ NK cells is associated with tumor invasion, lymph node metastasis, and the clinical stage (119). According to the study by Zheng et al. (119), TIM-3^+^ NK cells may be a potential prognostic factor for patients with EC. Additionally, some studies have shown that cytokines such as interleukin (IL)-4, transforming growth factor (TGF)-β, and IL-6 are capable of inducing the expression of TIM-3 in HCC cells and TIM-3 accelerates tumor growth by the auto-secretion of IL-6 (120–122). For autoimmune diseases, the situation is completely different. The anti-TIM-3 antibody was shown to exacerbate experimental autoimmune encephalomyelitis (EAE), which serves as an animal model for multiple sclerosis (MS). Studies using TIM-3-deficient mice and wild-type mice treated with the TIM-3-Ig fusion protein showed that TIM-3 signaling is required for the induction of antigen-specific tolerance and that TIM-3 blockade enhances the development of spontaneous autoimmunity (59, 123). Recent studies have shown that patients with TIM-3^+^PD-1^+^CD8^low^ have the worst RFS and OS. Conversely, patients with TIM-3^−^PD-1^−^CD8 high had the best RFS and OS (86). Furthermore, it was demonstrated by Zhou et al. (124) that TIM-3^+^ PD-1^+^ T cells show the lowest level of granzyme B, IFN-γ, and TNF-α, which indicates that TIM-3 and PD-1 are important for the suppression of T cells. Furthermore, Zhong et al. (125) suggested that TIM-3^+^ Foxp3^+^ Treg (TFT) cells are highly enriched in the TME of diffuse large B-cell lymphoma (DLBCL) and the abundance of TFT cells is correlated with a poor prognosis in patients with DLBCL, while the TIM-3 antibody is capable of inhibiting IL-10 secretion.

Zhao et al. (88) found that the high densities of PD-1^+^ and TIGIT^+^ tumor-infiltrating lymphocytes (TILs) were expressed in 47.4% and 49.4% of ESCC patients, respectively. In addition, CD155 and TIGIT are highly expressed in patients with primary small cell carcinoma of the esophagus and are associated with shorter OS and PFS, supporting their role as a prognostic biomarker. PD-1 and TIM-3 expression on CD4^+^ T cells was closely associated with the clinicopathological characteristics of patients with EC (89). Xie et al. (87) found that the expressions of PD-1, TIM-3, and TIGIT were upregulated in TILs, which could be associated with TIL exhaustion. It may indicate that the coinhibitory receptors PD-1, TIM-3, and TIGIT may be potential therapeutic oncotargets for EC (**Table 1**).

Wang et al. (126) found that increased co-expression of PD-L1 and TIGIT is associated with poor OS in patients with ESCC. Chiu et al. (127) found that PVRL1/TIGIT inhibitors could be developed for the treatment of HCC through animal experiments. Their experiments found that PVRL1 stabilizes cell surface PVR, which interacts with TIGIT. This suppresses the anti-tumor immune response. The findings of Chauvin et al. support combinatorial immunotherapy with IL-15 and TIGIT blockade to promote the NK-cell-mediated destruction of MHC class I–deficient melanoma (128). Freed-Pastor et al. (129) identified the CD155/TIGIT axis as a key driver of immune evasion in pancreatic cancer, and combination immunotherapy (TIGIT/PD-1 co-blockade plus CD40 agonism) can induce antitumor responses in preclinical models. Furthermore, the findings of He et al. support the candidacy of CD155/TIGIT as a potential therapeutic target in gastric cancer (130). Josefsson et al. (131) found that TIGIT blockade is a relevant strategy for improved immunotherapy in follicular lymphoma. Judge et al. (132) reported that combined IL-15 and TIGIT blockade may be a promising clinical strategy in soft tissue sarcoma. Kawashima et al. (133) also described that the TIGIT/CD155 axis mediates resistance to ICIs in patients with melanoma. Liu et al. (134) found that intratumor TIGIT^+^ CD8^+^ T cell abundance could serve as an independent prognosticator of clinical outcome and a predictive biomarker of inferior adjuvant chemotherapy responsiveness in muscle invasive bladder cancer. In Italy, Raphael et al. (135) described that IC pathways TIGIT and PD-1 are associated with patient outcome and antitumor immunity in glioblastoma.

## 4 Ongoing Clinical Trials in Esophageal Cancer

Current immunotherapy strategies are mainly focused on neoadjuvant immunotherapy combined with chemo/chemoradiotherapy and dual immuno-blockade. The PALACE-1 trial revealed that pembrolizumab combined with chemoradiotherapy was safe for patients with locally advanced ESCC (136). Likewise, neoadjuvant immunotherapy combined with concurrent chemoradiotherapy for locally advanced EC was shown to be safe and effective (137). The CheckMate 648 trial also showed that both nivolumab plus chemotherapy and nivolumab plus ipilimumab in first-line treatment displayed longer overall OS than chemotherapy alone for ESCC (138). The effectiveness of immunotherapy makes it a promising treatment in EC.

Recently, RELATIVITY-047 (NCT03470922), a phase-II/III double-blind and randomized trial comparing relatlimab plus nivolumab versus nivolumab in 714 patients with previously untreated, unresectable stage III or IV melanoma revealed that the median PFS was significantly higher in the relatlimab plus nivolumab group compared to that of the nivolumab group (10.1 months vs. 4.6 months, HR 0.75, P = 0.006) and there were no safety concerns. This study indicated that the dual blockade of LAG-3 and PD-1 provided better benefit in terms of PFS (139).

We also summarized and analyzed the current ongoing clinical trials in EC registered with clinicaltrials.gov evaluating LAG-3. A bispecific PD-1-LAG-3 antibody, RO7247669, has been tested in two clinical trials (NCT04140500 and NCT04785820). NCT04140500 was a phase I study to evaluate the safety/tolerability, pharmacokinetics, pharmacodynamics, and preliminary antitumor activity of RO7247669 in patients with various solid tumors, including ESCC. NCT04785820 was a phase II study to evaluate the safety and efficacy of RO7247669 (a PD-1-LAG-3 antibody) and RO7121661 (a PD-1-TIM-3 antibody), compared to nivolumab, in patients diagnosed with advanced or metastatic ESCC. These two studies are currently recruiting patients, so there are no results available. INCAGN02385 is an Fc-engineered IgG1κ antibody that targets LAG-3. INCAGN02385 has been tested in a wide range of solid tumors including HCC and EC (140, 141). NCT03538028 was a phase-I open-label dose-escalation trial to evaluate the safety, tolerability, and preliminary efficacy of INCAGN02385 in solid tumors, including EC, HCC, and lung cancer (141). The trial was completed, but no results are available.

NCT03708328 is another clinical trial evaluating RO7121661. It is an open-label, multicenter phase 1 multiple ascending dose study for participants with advanced and/or metastatic solid tumors but is not yet recruiting. Additionally, NCT03652077 is a study to determine the safety, tolerability, and preliminary efficacy of INCAGN02390 (a TIM-3 antibody) in participants with selected advanced malignancies. It is worth mentioning that INCAGN02390 also enhances IFN-γ production from T cells undergoing tonic TCR stimulation when combined with PD-1 blockade. The study was completed on November 15, 2021; however, the results have not yet been submitted. Furthermore, several studies have shown that the use of antibodies against PD-1 and TIM-3 in combination is also more effective than blocking TIM-3 alone in HCC (122, 124).

There are some clinical trials that have used tislelizumab and ociperlimab, atezolizumab and tiragolumab, or atezolizumab and tiragolumab to treat EC; however, they are all recruiting; thus, outcomes have not been reported to date (**Table 2**). Vibostolimab, an anti-TIGIT antibody, plus pembrolizumab, was well tolerated and has demonstrated antitumor activity in a phase I study for patients with advanced solid tumors, including advanced NSCLC (142). This study may provide a new landscape for the combination of anti-TIGIT and anti-PD-1 in EC treatment.

**Table 2:** The ongoing clinical trials evaluating ICIs targeting novel immune checkpoint pathways in EC.

Study design	Patient population	Status	Phase	Identifier			
**Arm A:** (single-agent dose escalation): RO7247669 (RG6139, PD1-LAG3 BsAb) **Arm B:** (tumor-specific expansion cohorts): RO7247669	Esophageal Squamous Cell Carcinoma* Solid tumors Metastatic melanoma Non-small cell lung cancer	Recruiting	Phase I	NCT04140500
**Arm A:** INCAGN02385	Gastric Cancer (Including Stomach and Gastroesophageal Junction* Esophageal Cancer* Cervical Cancer Microsatellite Instability (MSI)-High Endometrial Cancer Hepatocellular Carcinoma Melanoma (Uveal Melanoma Excluded) Merkel Cell Carcinoma Mesothelioma MSI-High Colorectal Cancer Non-Small Cell Lung Cancer Ovarian Cancer Squamous Cell Carcinoma of the Head and Neck Small Cell Lung Cancer Renal Cell Carcinoma Triple-Negative Breast Cancer Urothelial Carcinoma Diffuse Large B-Cell Lymphoma	Completed	Phase I	NCT03538028
**Arm A:** RO7121661 (RG7769, PD1-TIM-3 BsAb) **Arm B:** RO7247669 **Arm C:** Nivolumab (Opdivo, anti-PD-1 mAb)	Advanced or Metastatic Esophageal Squamous Cell Carcinoma*	Recruiting	Phase II	NCT04785820
**Arm A:** INCAGN02390 (anti-TIM-3)	Esophageal Cancer* Cervical Cancer Gastric Cancer Stomach Cancer Gastroesophageal Junction Cancer Hepatocellular Carcinoma Melanoma Uveal Melanoma Merkel Cell Carcinoma Mesothelioma MSI Non-Small Cell Lung Cancer Ovarian Cancer Squamous Cell Carcinoma of the Head and Neck Small Cell Lung Cancer Renal Cell Carcinoma Triple-Negative Breast Cancer Urothelial Carcinoma Mismatch Repair Deficiency	Active, not recruiting	Phase I	NCT03652077
**Arm A:** RO7121661	Esophageal Squamous Cell Carcinoma* Solid Tumors Metastatic Melanoma Non-Small Cell Lung Cancer Small Cell Lung Cancer	Recruiting	Phase I	NCT03708328
**Arm A:** Tislelizumab (anti-PD-1mAb) + Ociperlimab (Anti-TIGIT mAb) **Arm B:** Tislelizumab plus Placebo	Esophageal Squamous Cell Carcinoma*	Recruiting	Phase II	NCT04732494
**Arm A:** RO5541267 (Atezolizumab, anti-PD-L1mab) + RO7092284 (Tiragolumab, Anti-TIGITmAb) + Cisplatin + 5FU **Arm B:** RO5541267 + Cisplatin + 5-FU **Arm C:** Cisplatin + 5-FU **Arm D:** RO5541267 + RO7092284	Gastroesophageal Junction Adenocarcinoma* Esophageal Carcinoma* Gastric Adenocarcinoma	Recruiting	Phase II	NCT03281369
**Arm A:** RO7092284 + RO5541267 **Arm B:** RO7092284 Placebo + RO5541267 **Arm C:** RO7092284 Placebo + RO5541267 Placebo	Esophageal Squamous Cell Carcinoma*	Recruiting	Phase III	NCT04543617

*EC-related diseases. MSI, microsatellite instability.

## 5 Conclusions and Future Perspectives

ICs play a significant role in maintaining self-tolerance and preventing the occurrence of autoimmune diseases. Cancer cells can hijack these molecules, leading to T-cell exhaustion and dysfunction. Recent years have witnessed the rapid development of ICI-based immunotherapy, and novel ICIs are under investigation due to the limited efficacy and dose-dependent toxicity of the previous agents. LAG-3, TIM-3, and TIGIT have shown promising preclinical outcomes in a single agent trial, especially in collaboration with the PD-1 inhibitor.

Not all patients can benefit from immunotherapy; therefore, predicting patients who are prone to resistance to ICIs *via* biomarkers is of the essence to switch to alternative treatments (143). However, as recommended by the National Comprehensive Cancer Network, the feasibility of microsatellite instability (MSI), tumor mutational burden (TMB), and PD-L1 as biomarkers for patients after ICI still needs further elucidation (144, 145). Thus, exploring reliable biomarkers is of the essence. Shen et al. (146) demonstrated that patients with melanoma with an LAG immunotype (75.8 months) showed significantly longer median survival compared to patients with an LAG^+^ immunotype (22.2 months) (*P* = 0.031). However, the role of LAG-3 as a biomarker in EC is rarely reported. Our work supports the rationality of LAG-3 as a potential biomarker predicting prognosis in EC. Two questions emerge when considering ICs as reliable biomarkers: First, will LAG-3, sLAG-3, TIM-3, and TIGIT be able to predict benefits to other types of immunotherapy or conventional treatments, or even tumor progression? Second, what types of biomarkers are rational to assess therapeutic effects after receiving ICIs targeting LAG-3, TIM-3, or TIGIT? In summary, the use of precise biomarkers will lead to the development of precise personalized immunotherapies.

According to a model proposed by Anderson et al. (59), the impact of ICs on maintaining homeostasis is proportional to the autoimmune toxicity achieved after blocking. Based on this principle, LAG-3, TIM-3, and TIGIT rank second in the hierarchy with less toxicity, while CTLA-4 ranks the top of the model with the highest toxicity. This theory indicates that ICIs targeting the next generation of ICs may be safer than traditional ICIs targeting PD-1 and CTLA-4. In addition, studies on other newly emerging ICs are presented. VISTA, B7-H3, BTLA, and Siglec-15 should be examined in EC, so as to provide additional treatment strategies for patients at advanced stage.

Chemotherapy and radiation therapy can induce tumor cell death, thereby promoting the release of antigens that activate APCs (147). This process is called immunogenic cell death (147). The recruitment and infiltration of cytotoxic T lymphocytes are also observed after the treatment of chemotherapy and radiotherapy (148, 149). To elicit robust antitumor immunity, chemotherapy and radiation therapy can be combined with immunotherapy. Currently, the implementation of ICI focuses on the combination with other therapeutic strategies including radiotherapy and chemotherapy in EC, and substantial clinical trials have obtained encouraging results (5, 136, 138). Currently, a hotspot of LAG-3, TIM-3, and TIGIT involves the dual-blockade therapy with PD-1 because of their dramatic synergy with PD-1 (139, 142). To our knowledge, the effects of novel immunotherapy pathways with conventional treatment have not been determined. Therefore, future research should focus on the combination of ICIs and other treatment strategies, such as chemotherapy and radiation therapy, with the aim of broadening therapeutic options for advanced patients with EC.

## Author Contributions

XZ, TR, and HZ were responsible for the primary review of literature, the consolidation of information, and writing. CH and XG guided and supervised this study. All authors contributed to the article and approved the submitted version.

## Funding

This work was supported by grants from the National Natural Science Foundation of China [81901660], and China Scholarship Council [201808330646].

## Conflict of Interest

The authors declare that the research was conducted in the absence of any commercial or financial relationships that could be construed as a potential conflict of interest.

## Publisher’s Note

All claims expressed in this article are solely those of the authors and do not necessarily represent those of their affiliated organizations, or those of the publisher, the editors and the reviewers. Any product that may be evaluated in this article, or claim that may be made by its manufacturer, is not guaranteed or endorsed by the publisher.

## References

[1] Bray F Ferlay J Soerjomataram I Siegel RL Torre LA Jemal A. Global Cancer Statistics 2018: GLOBOCAN Estimates of Incidence and Mortality Worldwide for 36 Cancers in 185 Countries. CA Cancer J Clin (2018) 68(6):394–424. doi: 10.3322/caac.21492 30207593

[2] Abnet CC Arnold M Wei WQ. Epidemiology of Esophageal Squamous Cell Carcinoma. Gastroenterology (2018) 154(2):360–73. doi: 10.1053/j.gastro.2017.08.023 PMC5836473 28823862

[3] Yang YM Hong P Xu WW He QY Li B. Advances in Targeted Therapy for Esophageal Cancer. Signal Transduct Target Ther (2020) 5(1):229. doi: 10.1038/s41392-020-00323-3 33028804 PMC7542465

[4] Zhang L Ma J Han Y Liu J Zhou W Hong L. Targeted Therapy in Esophageal Cancer. Expert Rev Gastroenterol Hepatol (2016) 10(5):595–604. doi: 10.1586/17474124.2016.1140036 26895097

[5] Sun JM Shen L Shah MA Enzinger P Adenis A Doi T. Pembrolizumab Plus Chemotherapy Versus Chemotherapy Alone for First-Line Treatment of Advanced Oesophageal Cancer (KEYNOTE-590): A Randomised, Placebo-Controlled, Phase 3 Study. Lancet (2021) 398(10302):759–71. doi: 10.1016/s0140-6736(21)01234-4 34454674

[6] Kojima T Shah MA Muro K Francois E Adenis A Hsu CH. Randomized Phase III KEYNOTE-181 Study of Pembrolizumab Versus Chemotherapy in Advanced Esophageal Cancer. J Clin Oncol (2020) 38(35):4138–48. doi: 10.1200/jco.20.01888 33026938

[7] Andrews LP Yano H Vignali DAA. Inhibitory Receptors and Ligands Beyond PD-1, PD-L1 and CTLA-4: Breakthroughs or Backups. Nat Immunol (2019) 20(11):1425–34. doi: 10.1038/s41590-019-0512-0 31611702

[8] Qin S Xu L Yi M Yu S Wu K Luo S. Novel Immune Checkpoint Targets: Moving Beyond PD-1 and CTLA-4. Mol Cancer (2019) 18(1):155. doi: 10.1186/s12943-019-1091-2 31690319 PMC6833286

[9] Yuan Y Adam A Zhao C Chen H. Recent Advancements in the Mechanisms Underlying Resistance to PD-1/PD-L1 Blockade Immunotherapy. Cancers (Basel) (2021) 13(4):1–18. doi: 10.3390/cancers13040663 PMC7915065 33562324

[10] Baba Y Nomoto D Okadome K Ishimoto T Iwatsuki M Miyamoto Y. Tumor Immune Microenvironment and Immune Checkpoint Inhibitors in Esophageal Squamous Cell Carcinoma. Cancer Sci (2020) 111(9):3132–41. doi: 10.1111/cas.14541 PMC7469863 32579769

[11] Schnell A Bod L Madi A Kuchroo VK. The Yin and Yang of Co-Inhibitory Receptors: Toward Anti-Tumor Immunity Without Autoimmunity. Cell Res (2020) 30(4):285–99. doi: 10.1038/s41422-020-0277-x PMC7118128 31974523

[12] Chauvin JM Zarour HM. TIGIT in Cancer Immunotherapy. J Immunother Cancer (2020) 8(2):1–7. doi: 10.1136/jitc-2020-000957 PMC7477968 32900861

[13] Wolf Y Anderson AC Kuchroo VK. TIM3 Comes of Age as an Inhibitory Receptor. Nat Rev Immunol (2020) 20(3):173–85. doi: 10.1038/s41577-019-0224-6 PMC7327798 31676858

[14] Ruffo E Wu RC Bruno TC Workman CJ Vignali DAA. Lymphocyte-Activation Gene 3 (LAG3): The Next Immune Checkpoint Receptor. Semin Immunol (2019) 42:101305. doi: 10.1016/j.smim.2019.101305 31604537 PMC6920665

[15] Hung AL Maxwell R Theodros D Belcaid Z Mathios D Luksik AS. TIGIT and PD-1 Dual Checkpoint Blockade Enhances Antitumor Immunity and Survival in GBM. Oncoimmunology (2018) 7(8):e1466769. doi: 10.1080/2162402x.2018.1466769 30221069 PMC6136875

[16] Woo SR Turnis ME Goldberg MV Bankoti J Selby M Nirschl CJ. Immune Inhibitory Molecules LAG-3 and PD-1 Synergistically Regulate T-Cell Function to Promote Tumoral Immune Escape. Cancer Res (2012) 72(4):917–27. doi: 10.1158/0008-5472.Can-11-1620 PMC3288154 22186141

[17] Sakuishi K Apetoh L Sullivan JM Blazar BR Kuchroo VK Anderson AC. Targeting Tim-3 and PD-1 Pathways to Reverse T Cell Exhaustion and Restore Anti-Tumor Immunity. J Exp Med (2010) 207(10):2187–94. doi: 10.1084/jem.20100643 PMC2947065 20819927

[18] Puhr HC Ilhan-Mutlu A. New Emerging Targets in Cancer Immunotherapy: The Role of LAG3. ESMO Open (2019) 4(2):e000482. doi: 10.1136/esmoopen-2018-000482 31231559 PMC6555869

[19] Melaiu O Lucarini V Giovannoni R Fruci D Gemignani F. News on Immune Checkpoint Inhibitors as Immunotherapy Strategies in Adult and Pediatric Solid Tumors. Semin Cancer Biol (2020) 79:18–43. doi: 10.1016/j.semcancer.2020.07.001 32659257

[20] Shan C Li X Zhang J. Progress of Immune Checkpoint LAG-3 in Immunotherapy. Oncol Lett (2020) 20(5):207. doi: 10.3892/ol.2020.12070 32963613 PMC7491111

[21] Andrews LP Marciscano AE Drake CG Vignali DA. LAG3 (CD223) as a Cancer Immunotherapy Target. Immunol Rev (2017) 276(1):80–96. doi: 10.1111/imr.12519 28258692 PMC5338468

[22] Maruhashi T Sugiura D Okazaki IM Okazaki T. LAG-3: From Molecular Functions to Clinical Applications. J Immunother Cancer (2020) 8(2):1–9. doi: 10.1136/jitc-2020-001014 PMC7488795 32929051

[23] Li N Wang Y Forbes K Vignali KM Heale BS Saftig P. Metalloproteases Regulate T-Cell Proliferation and Effector Function *via* LAG-3. EMBO J (2007) 26(2):494–504. doi: 10.1038/sj.emboj.7601520 17245433 PMC1783452

[24] Hu S Liu X Li T Li Z Hu F. LAG3 (CD223) and Autoimmunity: Emerging Evidence. J Autoimmun (2020) 112:102504. doi: 10.1016/j.jaut.2020.102504 32576412

[25] Maeda TK Sugiura D Okazaki IM Maruhashi T Okazaki T. Atypical Motifs in the Cytoplasmic Region of the Inhibitory Immune Co-Receptor LAG-3 Inhibit T Cell Activation. J Biol Chem (2019) 294(15):6017–26. doi: 10.1074/jbc.RA119.007455 PMC6463702 30760527

[26] Workman CJ Vignali DA. The CD4-Related Molecule, LAG-3 (CD223), Regulates the Expansion of Activated T Cells. Eur J Immunol (2003) 33(4):970–9. doi: 10.1002/eji.200323382 12672063

[27] Workman CJ Dugger KJ Vignali DA. Cutting Edge: Molecular Analysis of the Negative Regulatory Function of Lymphocyte Activation Gene-3. J Immunol (2002) 169(10):5392–5. doi: 10.4049/jimmunol.169.10.5392 12421911

[28] Iouzalen N Andreae S Hannier S Triebel F. LAP, a Lymphocyte Activation Gene-3 (LAG-3)-Associated Protein That Binds to a Repeated EP Motif in the Intracellular Region of LAG-3, may Participate in the Down-Regulation of the CD3/TCR Activation Pathway. Eur J Immunol (2001) 31(10):2885–91. doi: 10.1002/1521-4141(2001010)31:10<2885::AID-IMMU2885>3.0.CO;2-2 11592063

[29] Durham NM Nirschl CJ Jackson CM Elias J Kochel CM Anders RA. Lymphocyte Activation Gene 3 (LAG-3) Modulates the Ability of CD4 T-Cells to be Suppressed *In Vivo* . PloS One (2014) 9(11):e109080. doi: 10.1371/journal.pone.0109080 25372844 PMC4220939

[30] Müller MR Rao A. NFAT, Immunity and Cancer: A Transcription Factor Comes of Age. Nat Rev Immunol (2010) 10(9):645–56. doi: 10.1038/nri2818 20725108

[31] Dienz O Eaton SM Krahl TJ Diehl S Charland C Dodge J. Accumulation of NFAT Mediates IL-2 Expression in Memory, But Not Naïve, CD4+ T Cells. Proc Natl Acad Sci USA (2007) 104(17):7175–80. doi: 10.1073/pnas.0610442104 PMC1855411 17438271

[32] Wang H Kaur G Sankin AI Chen F Guan F Zang X. Immune Checkpoint Blockade and CAR-T Cell Therapy in Hematologic Malignancies. J Hematol Oncol (2019) 12(1):59. doi: 10.1186/s13045-019-0746-1 31186046 PMC6558778

[33] Mei Z Huang J Qiao B Lam AK. Immune Checkpoint Pathways in Immunotherapy for Head and Neck Squamous Cell Carcinoma. Int J Oral Sci (2020) 12(1):16. doi: 10.1038/s41368-020-0084-8 32461587 PMC7253444

[34] Angelopoulou E Paudel YN Villa C Shaikh MF Piperi C. Lymphocyte-Activation Gene 3 (LAG3) Protein as a Possible Therapeutic Target for Parkinson's Disease: Molecular Mechanisms Connecting Neuroinflammation to α-Synuclein Spreading Pathology. Biol (Basel) (2020) 9(4).:1–13 doi: 10.3390/biology9040086 PMC7235703 32340360

[35] Lecocq Q Keyaerts M Devoogdt N Breckpot K. The Next-Generation Immune Checkpoint LAG-3 and Its Therapeutic Potential in Oncology: Third Time's a Charm. Int J Mol Sci (2020) 22(1):1–17. doi: 10.3390/ijms22010075 PMC7795594 33374804

[36] Wang W Chen D Zhao Y Zhao T Wen J Mao Y. Characterization of LAG-3, CTLA-4, and CD8(+) TIL Density and Their Joint Influence on the Prognosis of Patients With Esophageal Squamous Cell Carcinoma. Ann Transl Med (2019) 7(23):776. doi: 10.21037/atm.2019.11.38 32042792 PMC6990025

[37] Triebel F Jitsukawa S Baixeras E Roman-Roman S Genevee C Viegas-Pequignot E. LAG-3, a Novel Lymphocyte Activation Gene Closely Related to CD4. J Exp Med (1990) 171(5):1393–405. doi: 10.1084/jem.171.5.1393 PMC2187904 1692078

[38] Demeure CE Wolfers J Martin-Garcia N Gaulard P Triebel F. T Lymphocytes Infiltrating Various Tumour Types Express the MHC Class II Ligand Lymphocyte Activation Gene-3 (LAG-3): Role of LAG-3/MHC Class II Interactions in Cell-Cell Contacts. Eur J Cancer (2001) 37(13):1709–18. doi: 10.1016/s0959-8049(01)00184-8 11527700

[39] Solinas C Migliori E De Silva P Willard-Gallo K. LAG3: The Biological Processes That Motivate Targeting This Immune Checkpoint Molecule in Human Cancer. Cancers (Basel) (2019) 11(8):1–16. doi: 10.3390/cancers11081213 PMC6721578 31434339

[40] Shin DS Ribas A. The Evolution of Checkpoint Blockade as a Cancer Therapy: What's Here, What's Next? Curr Opin Immunol (2015) 33:23–35. doi: 10.1016/j.coi.2015.01.006 25621841

[41] Workman CJ Cauley LS Kim IJ Blackman MA Woodland DL Vignali DA. Lymphocyte Activation Gene-3 (CD223) Regulates the Size of the Expanding T Cell Population Following Antigen Activation *In Vivo* . J Immunol (2004) 172(9):5450–5. doi: 10.4049/jimmunol.172.9.5450 15100286

[42] Chou FC Chen HY Kuo CC Sytwu HK. Role of Galectins in Tumors and in Clinical Immunotherapy. Int J Mol Sci (2018) 19(2):1–11. doi: 10.3390/ijms19020430 PMC5855652 29389859

[43] Ahmed H AlSadek DM. Galectin-3 as a Potential Target to Prevent Cancer Metastasis. Clin Med Insights Oncol (2015) 9:113–21. doi: 10.4137/cmo.S29462 PMC4662425 26640395

[44] Thijssen VL Heusschen R Caers J Griffioen AW. Galectin Expression in Cancer Diagnosis and Prognosis: A Systematic Review. Biochim Biophys Acta (2015) 1855(2):235–47. doi: 10.1016/j.bbcan.2015.03.003 25819524

[45] Kouo T Huang L Pucsek AB Cao M Solt S Armstrong T. Galectin-3 Shapes Antitumor Immune Responses by Suppressing CD8+ T Cells *via* LAG-3 and Inhibiting Expansion of Plasmacytoid Dendritic Cells. Cancer Immunol Res (2015) 3(4):412–23. doi: 10.1158/2326-6066.Cir-14-0150 PMC4390508 25691328

[46] Liu D Lu Q Wang X Wang J Lu N Jiang Z. LSECtin on Tumor-Associated Macrophages Enhances Breast Cancer Stemness *via* Interaction With its Receptor BTN3A3. Cell Res (2019) 29(5):365–78. doi: 10.1038/s41422-019-0155-6 PMC6796923 30858559

[47] Xu F Liu J Liu D Liu B Wang M Hu Z. LSECtin Expressed on Melanoma Cells Promotes Tumor Progression by Inhibiting Antitumor T-Cell Responses. Cancer Res (2014) 74(13):3418–28. doi: 10.1158/0008-5472.Can-13-2690 24769443

[48] Zhang WT Liu TT Wu M Chen XC Han L Shi ZZ. Development of a Nanobody-Based Immunoassay for the Sensitive Detection of Fibrinogen-Like Protein 1. Acta Pharmacol Sin (2021) 42(11):1921–9. doi: 10.1038/s41401-020-00574-4 PMC8563870 33633363

[49] Wang J Sanmamed MF Datar I Su TT Ji L Sun J. Fibrinogen-Like Protein 1 Is a Major Immune Inhibitory Ligand of LAG-3. Cell (2019) 176(1-2):334–47.e12. doi: 10.1016/j.cell.2018.11.010 30580966 PMC6365968

[50] Mao X Ou MT Karuppagounder SS Kam TI Yin X Xiong Y. Pathological α-Synuclein Transmission Initiated by Binding Lymphocyte-Activation Gene 3. Science (2016) 353(6307):aah3374–aah3374. doi: 10.1126/science.aah3374 PMC5510615 27708076

[51] Das M Zhu C Kuchroo VK. Tim-3 and its Role in Regulating Anti-Tumor Immunity. Immunol Rev (2017) 276(1):97–111. doi: 10.1111/imr.12520 28258697 PMC5512889

[52] Joller N Kuchroo VK. Tim-3, Lag-3, and TIGIT. Curr Top Microbiol Immunol (2017) 410:127–56. doi: 10.1007/82_2017_62 PMC5902028 28900677

[53] Zhu C Anderson AC Schubart A Xiong H Imitola J Khoury SJ. The Tim-3 Ligand Galectin-9 Negatively Regulates T Helper Type 1 Immunity. Nat Immunol (2005) 6(12):1245–52. doi: 10.1038/ni1271 16286920

[54] Tang R Rangachari M Kuchroo VK. Tim-3: A Co-Receptor With Diverse Roles in T Cell Exhaustion and Tolerance. Semin Immunol (2019) 42:101302. doi: 10.1016/j.smim.2019.101302 31604535

[55] Kanzaki M Wada J Sugiyama K Nakatsuka A Teshigawara S Murakami K. Galectin-9 and T Cell Immunoglobulin Mucin-3 Pathway is a Therapeutic Target for Type 1 Diabetes. Endocrinology (2012) 153(2):612–20. doi: 10.1210/en.2011-1579 22186414

[56] Yang L Anderson DE Kuchroo J Hafler DA. Lack of TIM-3 Immunoregulation in Multiple Sclerosis. J Immunol (2008) 180(7):4409–14. doi: 10.4049/jimmunol.180.7.4409 18354161

[57] Zhang Y Ma CJ Wang JM Ji XJ Wu XY Jia ZS. Tim-3 Negatively Regulates IL-12 Expression by Monocytes in HCV Infection. PloS One (2011) 6(5):e19664. doi: 10.1371/journal.pone.0019664 21637332 PMC3102652

[58] Zhang W Zhang Y He Y Wang X Fang Q. Lipopolysaccharide Mediates Time-Dependent Macrophage M1/M2 Polarization Through the Tim-3/Galectin-9 Signalling Pathway. Exp Cell Res (2019) 376(2):124–32. doi: 10.1016/j.yexcr.2019.02.007 30763585

[59] Anderson AC Joller N Kuchroo VK. Lag-3, Tim-3, and TIGIT: Co-Inhibitory Receptors With Specialized Functions in Immune Regulation. Immunity (2016) 44(5):989–1004. doi: 10.1016/j.immuni.2016.05.001 27192565 PMC4942846

[60] Lee J Su EW Zhu C Hainline S Phuah J Moroco JA. Phosphotyrosine-Dependent Coupling of Tim-3 to T-Cell Receptor Signaling Pathways. Mol Cell Biol (2011) 31(19):3963–74. doi: 10.1128/MCB.05297-11 PMC3187355 21807895

[61] van de Weyer PS Muehlfeit M Klose C Bonventre JV Walz G Kuehn EW. A Highly Conserved Tyrosine of Tim-3 is Phosphorylated Upon Stimulation by its Ligand Galectin-9. Biochem Biophys Res Commun (2006) 351(2):571–6. doi: 10.1016/j.bbrc.2006.10.079 17069754

[62] Rangachari M Zhu C Sakuishi K Xiao S Karman J Chen A. Bat3 Promotes T Cell Responses and Autoimmunity by Repressing Tim-3–Mediated Cell Death and Exhaustion. Nat Med (2012) 18(9):1394–400. doi: 10.1038/nm.2871 PMC3491118 22863785

[63] Huang YH Zhu C Kondo Y Anderson AC Gandhi A Russell A. CEACAM1 Regulates TIM-3-Mediated Tolerance and Exhaustion. Nature (2015) 517(7534):386–90. doi: 10.1038/nature13848 PMC4297519 25363763

[64] Davidson D Schraven B Veillette A. PAG-Associated FynT Regulates Calcium Signaling and Promotes Anergy in T Lymphocytes. Mol Cell Biol (2007) 27(5):1960–73. doi: 10.1128/MCB.01983-06 PMC1820463 17210649

[65] Salmond RJ Filby A Qureshi I Caserta S Zamoyska R. T-Cell Receptor Proximal Signaling *via* the Src-Family Kinases, Lck and Fyn, Influences T-Cell Activation, Differentiation, and Tolerance. Immunol Rev (2009) 228(1):9–22. doi: 10.1111/j.1600-065X.2008.00745.x 19290918

[66] Smida M Posevitz-Fejfar A Horejsi V Schraven B Lindquist JA. A Novel Negative Regulatory Function of the Phosphoprotein Associated With Glycosphingolipid-Enriched Microdomains: Blocking Ras Activation. Blood (2007) 110(2):596–615. doi: 10.1182/blood-2006-07-038752 17389760

[67] Zhou X Zuo C Li W Shi W Zhou X Wang H. A Novel D-Peptide Identified by Mirror-Image Phage Display Blocks TIGIT/PVR for Cancer Immunotherapy. Angew Chem Int Ed Engl (2020) 59(35):15114–8. doi: 10.1002/anie.202002783 32386245

[68] Zhang Q Bi J Zheng X Chen Y Wang H Wu W. Blockade of the Checkpoint Receptor TIGIT Prevents NK Cell Exhaustion and Elicits Potent Anti-Tumor Immunity. Nat Immunol (2018) 19(7):723–32. doi: 10.1038/s41590-018-0132-0 29915296

[69] Sasidharan Nair V Elkord E. Immune Checkpoint Inhibitors in Cancer Therapy: A Focus on T-Regulatory Cells. Immunol Cell Biol (2018) 96(1):21–33. doi: 10.1111/imcb.1003 29359507

[70] Yu X Harden K Gonzalez LC Francesco M Chiang E Irving B. The Surface Protein TIGIT Suppresses T Cell Activation by Promoting the Generation of Mature Immunoregulatory Dendritic Cells. Nat Immunol (2009) 10(1):48–57. doi: 10.1038/ni.1674 19011627

[71] Reches A Ophir Y Stein N Kol I Isaacson B Charpak Amikam Y. Nectin4 is a Novel TIGIT Ligand Which Combines Checkpoint Inhibition and Tumor Specificity. J Immunother Cancer (2020) 8(1):1–9. doi: 10.1136/jitc-2019-000266 PMC7279670 32503945

[72] Harjunpää H Guillerey C. TIGIT as an Emerging Immune Checkpoint. Clin Exp Immunol (2020) 200(2):108–19. doi: 10.1111/cei.13407 PMC7160651 31828774

[73] Whelan S Ophir E Kotturi MF Levy O Ganguly S Leung L. PVRIG and PVRL2 Are Induced in Cancer and Inhibit CD8(+) T-Cell Function. Cancer Immunol Res (2019) 7(2):257–68. doi: 10.1158/2326-6066.Cir-18-0442 PMC7001734 30659054

[74] Weulersse M Asrir A Pichler AC Lemaitre L Braun M Carrié N. Eomes-Dependent Loss of the Co-Activating Receptor CD226 Restrains CD8(+) T Cell Anti-Tumor Functions and Limits the Efficacy of Cancer Immunotherapy. Immunity (2020) 53(4):824–39.e10. doi: 10.1016/j.immuni.2020.09.006 33053331

[75] Pietra G Mingari MC Moretta L. TIGIT Blockade and IL15 in Tumor Immunotherapy: Together is Better. Clin Cancer Res (2020) 26(20):5274–5. doi: 10.1158/1078-0432.Ccr-20-2538 32817077

[76] Huang X Zhang X Li E Zhang G Wang X Tang T. VISTA: An Immune Regulatory Protein Checking Tumor and Immune Cells in Cancer Immunotherapy. J Hematol Oncol (2020) 13(1):83. doi: 10.1186/s13045-020-00917-y 32600443 PMC7325042

[77] Flem-Karlsen K Fodstad Ø Tan M Nunes-Xavier CE. B7-H3 in Cancer - Beyond Immune Regulation. Trends Cancer (2018) 4(6):401–4. doi: 10.1016/j.trecan.2018.03.010 29860983

[78] Chen YL Lin HW Chien CL Lai YL Sun WZ Chen CA. BTLA Blockade Enhances Cancer Therapy by Inhibiting IL-6/IL-10-Induced CD19(high) B Lymphocytes. J Immunother Cancer (2019) 7(1):313. doi: 10.1186/s40425-019-0744-4 31753019 PMC6868712

[79] Wang J Sun J Liu LN Flies DB Nie X Toki M. Siglec-15 as an Immune Suppressor and Potential Target for Normalization Cancer Immunotherapy. Nat Med (2019) 25(4):656–66. doi: 10.1038/s41591-019-0374-x PMC7175920 30833750

[80] Nowak EC Lines JL Varn FS Deng J Sarde A Mabaera R. Immunoregulatory Functions of VISTA. Immunol Rev (2017) 276(1):66–79. doi: 10.1111/imr.12525 28258694 PMC5702497

[81] Wang J Wu G Manick B Hernandez V Renelt M Erickson C. VSIG-3 as a Ligand of VISTA Inhibits Human T-Cell Function. Immunology (2019) 156(1):74–85. doi: 10.1111/imm.13001 30220083 PMC6283650

[82] Mulati K Hamanishi J Matsumura N Chamoto K Mise N Abiko K. VISTA Expressed in Tumour Cells Regulates T Cell Function. Br J Cancer (2019) 120(1):115–27. doi: 10.1038/s41416-018-0313-5 PMC6325144 30382166

[83] Loeser H Kraemer M Gebauer F Bruns C Schröder W Zander T. The Expression of the Immune Checkpoint Regulator VISTA Correlates With Improved Overall Survival in Pt1/2 Tumor Stages in Esophageal Adenocarcinoma. Oncoimmunology (2019) 8(5):e1581546. doi: 10.1080/2162402x.2019.1581546 31069143 PMC6492979

[84] Gebauer F Krämer M Bruns C Schlößer HA Thelen M Lohneis P. Lymphocyte Activation Gene-3 (LAG3) mRNA and Protein Expression on Tumour Infiltrating Lymphocytes (TILs) in Oesophageal Adenocarcinoma. J Cancer Res Clin Oncol (2020) 146(9):2319–27. doi: 10.1007/s00432-020-03295-7 PMC7382658 32592066

[85] Zhang Y Liu YD Luo YL Liu BL Huang QT Wang F. Prognostic Value of Lymphocyte Activation Gene-3 (LAG-3) Expression in Esophageal Squamous Cell Carcinoma. J Cancer (2018) 9(22):4287–93. doi: 10.7150/jca.26949 PMC6277627 30519331

[86] Zheng Y Li Y Lian J Yang H Li F Zhao S. TNF-Alpha-Induced Tim-3 Expression Marks the Dysfunction of Infiltrating Natural Killer Cells in Human Esophageal Cancer. J Transl Med (2019) 17(1):165. doi: 10.1186/s12967-019-1917-0 31109341 PMC6528366

[87] Xie J Wang J Cheng S Zheng L Ji F Yang L. Expression of Immune Checkpoints in T Cells of Esophageal Cancer Patients. Oncotarget (2016) 7(39):63669–78. doi: 10.18632/oncotarget.11611 PMC5325394 27577071

[88] Zhao JJ Zhou ZQ Wang P Chen CL Liu Y Pan QZ. Orchestration of Immune Checkpoints in Tumor Immune Contexture and Their Prognostic Significance in Esophageal Squamous Cell Carcinoma. Cancer Manag Res (2018) 10:6457–68. doi: 10.2147/cmar.S181949 PMC6276823 30568505

[89] Zhao K Ma L Feng L Huang Z Meng X Yu J. CD155 Overexpression Correlates With Poor Prognosis in Primary Small Cell Carcinoma of the Esophagus. Front Mol Biosci (2020) 7:608404. doi: 10.3389/fmolb.2020.608404 33490104 PMC7817973

[90] Chen L Chen J Xu B Wang Q Zhou W Zhang G. B7-H3 Expression Associates With Tumor Invasion and Patient's Poor Survival in Human Esophageal Cancer. Am J Transl Res (2015) 7(12):2646–60. PMC4731663 26885263

[91] Wang L Cao NN Wang S Man HW Li PF Shan BE. Roles of Coinhibitory Molecules B7-H3 and B7-H4 in Esophageal Squamous Cell Carcinoma. Tumour Biol (2016) 37(3):2961–71. doi: 10.1007/s13277-015-4132-5 26411671

[92] Chen Z Cao K Zhang J Liu Z Lu L Qi B. Concomitant Expression of Inhibitory Molecules for T Cell Activation Predicts Poor Survival in Patients With Esophageal Squamous Cell Carcinoma. Curr Cancer Drug Targets (2020) 21:244–53. doi: 10.2174/1568009620666201120152333 33222673

[93] Ceeraz S Nowak EC Noelle RJ. B7 Family Checkpoint Regulators in Immune Regulation and Disease. Trends Immunol (2013) 34(11):556–63. doi: 10.1016/j.it.2013.07.003 PMC3821798 23954143

[94] Gavrieli M Sedy J Nelson CA Murphy KM. BTLA and HVEM Cross Talk Regulates Inhibition and Costimulation. Adv Immunol (2006) 92:157–85. doi: 10.1016/s0065-2776(06)92004-5 17145304

[95] Ren X. Immunosuppressive Checkpoint Siglec-15: A Vital New Piece of the Cancer Immunotherapy Jigsaw Puzzle. Cancer Biol Med (2019) 16(2):205–10. doi: 10.20892/j.issn.2095-3941.2018.0141 PMC6713637 31516742

[96] Siglec-15: An Attractive Immunotherapy Target. Cancer Discov (2020) 10(1):7–8. doi: 10.1158/2159-8290.Cd-nb2019-136 31806628

[97] Sun J Lu Q Sanmamed MF Wang J. Siglec-15 as an Emerging Target for Next-Generation Cancer Immunotherapy. Clin Cancer Res (2021) 27(3):680–8. doi: 10.1158/1078-0432.Ccr-19-2925 PMC9942711 32958700

[98] Sanmamed MF Chen L. A Paradigm Shift in Cancer Immunotherapy: From Enhancement to Normalization. Cell (2018) 175(2):313–26. doi: 10.1016/j.cell.2018.09.035 PMC6538253 30290139

[99] Graydon CG Mohideen S Fowke KR. LAG3's Enigmatic Mechanism of Action. Front Immunol (2020) 11:615317. doi: 10.3389/fimmu.2020.615317 33488626 PMC7820757

[100] Yoshida J Ishikawa T Doi T Ota T Yasuda T Okayama T. Clinical Significance of Soluble Forms of Immune Checkpoint Molecules in Advanced Esophageal Cancer. Med Oncol (2019) 36(7):60. doi: 10.1007/s12032-019-1285-x 31134385

[101] Buisson S Triebel F. LAG-3 (CD223) Reduces Macrophage and Dendritic Cell Differentiation From Monocyte Precursors. Immunology (2005) 114(3):369–74. doi: 10.1111/j.1365-2567.2004.02087.x PMC1782096 15720438

[102] Annunziato F Manetti R Tomasévic I Guidizi MG Biagiotti R Giannò V. Expression and Release of LAG-3-Encoded Protein by Human CD4+ T Cells are Associated With IFN-Gamma Production. FASEB J (1996) 10(7):769–76. doi: 10.1096/fasebj.10.7.8635694 8635694

[103] Nguyen LT Ohashi PS. Clinical Blockade of PD1 and LAG3–potential Mechanisms of Action. Nat Rev Immunol (2015) 15(1):45–56. doi: 10.1038/nri3790 25534622

[104] Triebel F. LAG-3: A Regulator of T-Cell and DC Responses and its Use in Therapeutic Vaccination. Trends Immunol (2003) 24(12):619–22. doi: 10.1016/j.it.2003.10.001 14644131

[105] Wang S Yu P Meng Z Feng L. The Value of microRNA-203 as a Biomarker for the Prognosis of Esophageal Cancer: A Protocol for Systematic Review and Meta-Analysis. Med (Baltimore) (2020) 99(50):e23599. doi: 10.1097/md.0000000000023599 PMC7738148 33327325

[106] Guo M Yuan F Qi F Sun J Rao Q Zhao Z. Expression and Clinical Significance of LAG-3, FGL1, PD-L1 and CD8(+)T Cells in Hepatocellular Carcinoma Using Multiplex Quantitative Analysis. J Transl Med (2020) 18(1):306. doi: 10.1186/s12967-020-02469-8 32762721 PMC7409704

[107] Kamal AM Wasfey EF Elghamry WR Sabry OM Elghobary HA Radwan SM. Genetic Signature of CTLA-4, BTLA, TIM-3 and LAG-3 Molecular Expression in Colorectal Cancer Patients: Implications in Diagnosis and Survival Outcomes. Clin Biochem (2021) 96:13–8. doi: 10.1016/j.clinbiochem.2021.06.007 34217699

[108] Babar L Kosovec JE Jahangiri V Chowdhury N Zheng P Omstead AN. Prognostic Immune Markers for Recurrence and Survival in Locally Advanced Esophageal Adenocarcinoma. Oncotarget (2019) 10(44):4546–55. doi: 10.18632/oncotarget.27052 PMC6642049 31360303

[109] Sidaway P. Breast Cancer: LAG3 Expression Indicates Favourable Outcomes. Nat Rev Clin Oncol (2017) 14(12):712. doi: 10.1038/nrclinonc.2017.164 28994421

[110] Shi AP Tang XY Xiong YL Zheng KF Liu YJ Shi XG. Immune Checkpoint LAG3 and Its Ligand FGL1 in Cancer. Front Immunol (2021) 12:785091. doi: 10.3389/fimmu.2021.785091 35111155 PMC8801495

[111] Sobottka B Moch H Varga Z. Differential PD-1/LAG-3 Expression and Immune Phenotypes in Metastatic Sites of Breast Cancer. Breast Cancer Res (2021) 23(1):4. doi: 10.1186/s13058-020-01380-w 33413541 PMC7792100

[112] Wang Q Zhang J Tu H Liang D Chang DW Ye Y. Soluble Immune Checkpoint-Related Proteins as Predictors of Tumor Recurrence, Survival, and T Cell Phenotypes in Clear Cell Renal Cell Carcinoma Patients. J Immunother Cancer (2019) 7(1):334. doi: 10.1186/s40425-019-0810-y 31783776 PMC6884764

[113] Triebel F Hacene K Pichon MF. A Soluble Lymphocyte Activation Gene-3 (sLAG-3) Protein as a Prognostic Factor in Human Breast Cancer Expressing Estrogen or Progesterone Receptors. Cancer Lett (2006) 235(1):147–53. doi: 10.1016/j.canlet.2005.04.015 15946792

[114] Li N Jilisihan B Wang W Tang Y Keyoumu S. Soluble LAG3 Acts as a Potential Prognostic Marker of Gastric Cancer and its Positive Correlation With CD8+T Cell Frequency and Secretion of IL-12 and INF-γ in Peripheral Blood. Cancer Biomark (2018) 23(3):341–51. doi: 10.3233/cbm-181278 PMC13078572 30223387

[115] He Y Wang Y Zhao S Zhao C Zhou C Hirsch FR. sLAG-3 in non-Small-Cell Lung Cancer Patients' Serum. Onco Targets Ther (2018) 11:4781–4. doi: 10.2147/ott.S164178 PMC6097502 30147330

[116] Yu X Huang X Chen X Liu J Wu C Pu Q. Characterization of a Novel Anti-Human Lymphocyte Activation Gene 3 (LAG-3) Antibody for Cancer Immunotherapy. MAbs (2019) 11(6):1139–48. doi: 10.1080/19420862.2019.1629239 PMC6748621 31242068

[117] Balkwill F. Tumour Necrosis Factor and Cancer. Nat Rev Cancer (2009) 9(5):361–71. doi: 10.1038/nrc2628 19343034

[118] Yan W Liu X Ma H Zhang H Song X Gao L. Tim-3 Fosters HCC Development by Enhancing TGF-β-Mediated Alternative Activation of Macrophages. Gut (2015) 64(10):1593–604. doi: 10.1136/gutjnl-2014-307671 25608525

[119] Zheng Y Li Y Lian J Yang H Li F Zhao S. TNF-α-Induced Tim-3 Expression Marks the Dysfunction of Infiltrating Natural Killer Cells in Human Esophageal Cancer. J Transl Med (2019) 17(1):165. doi: 10.1186/s12967-019-1917-0 31109341 PMC6528366

[120] Zhang H Song Y Yang H Liu Z Gao L Liang X. Tumor Cell-Intrinsic Tim-3 Promotes Liver Cancer *via* NF-κb/IL-6/STAT3 Axis. Oncogene (2018) 37(18):2456–68. doi: 10.1038/s41388-018-0140-4 29449693

[121] Liu F Liu Y Chen Z. Tim-3 Expression and its Role in Hepatocellular Carcinoma. J Hematol Oncol (2018) 11(1):126. doi: 10.1186/s13045-018-0667-4 30309387 PMC6182863

[122] Ganjalikhani Hakemi M Jafarinia M Azizi M Rezaeepoor M Isayev O Bazhin AV. The Role of TIM-3 in Hepatocellular Carcinoma: A Promising Target for Immunotherapy? Front Oncol (2020) 10:601661. doi: 10.3389/fonc.2020.601661 33425759 PMC7793963

[123] Sabatos CA Chakravarti S Cha E Schubart A Sánchez-Fueyo A Zheng XX. Interaction of Tim-3 and Tim-3 Ligand Regulates T Helper Type 1 Responses and Induction of Peripheral Tolerance. Nat Immunol (2003) 4(11):1102–10. doi: 10.1038/ni988 14556006

[124] Zhou G Sprengers D Boor PPC Doukas M Schutz H Mancham S. Antibodies Against Immune Checkpoint Molecules Restore Functions of Tumor-Infiltrating T Cells in Hepatocellular Carcinomas. Gastroenterology (2017) 153(4):1107–19.e10. doi: 10.1053/j.gastro.2017.06.017 28648905

[125] Zhong W Liu X Zhu Z Li Q Li K. High Levels of Tim-3(+)Foxp3(+)Treg Cells in the Tumor Microenvironment is a Prognostic Indicator of Poor Survival of Diffuse Large B Cell Lymphoma Patients. Int Immunopharmacol (2021) 96:107662. doi: 10.1016/j.intimp.2021.107662 33864956

[126] Wang P Chen Y Long Q Li Q Tian J Liu T. Increased Coexpression of PD-L1 and TIM3/TIGIT is Associated With Poor Overall Survival of Patients With Esophageal Squamous Cell Carcinoma. J Immunother Cancer (2021) 9(10):1–11. doi: 10.1136/jitc-2021-002836 PMC8504357 34625514

[127] Chiu DK Yuen VW Cheu JW Wei LL Ting V Fehlings M. Hepatocellular Carcinoma Cells Up-Regulate PVRL1, Stabilizing PVR and Inhibiting the Cytotoxic T-Cell Response *via* TIGIT to Mediate Tumor Resistance to PD1 Inhibitors in Mice. Gastroenterology (2020) 159(2):609–23. doi: 10.1053/j.gastro.2020.03.074 32275969

[128] Chauvin JM Ka M Pagliano O Menna C Ding Q DeBlasio R. IL15 Stimulation With TIGIT Blockade Reverses CD155-Mediated NK-Cell Dysfunction in Melanoma. Clin Cancer Res (2020) 26(20):5520–33. doi: 10.1158/1078-0432.CCR-20-0575 PMC8045409 32591463

[129] Freed-Pastor WA Lambert LJ Ely ZA Pattada NB Bhutkar A Eng G. The CD155/TIGIT Axis Promotes and Maintains Immune Evasion in Neoantigen-Expressing Pancreatic Cancer. Cancer Cell (2021) 39(10):1342–60.e14. doi: 10.1016/j.ccell.2021.07.007 34358448 PMC8511341

[130] He W Zhang H Han F Chen X Lin R Wang W. CD155T/TIGIT Signaling Regulates CD8(+) T-Cell Metabolism and Promotes Tumor Progression in Human Gastric Cancer. Cancer Res (2017) 77(22):6375–88. doi: 10.1158/0008-5472.CAN-17-0381 28883004

[131] Josefsson SE Huse K Kolstad A Beiske K Pende D Steen CB. T Cells Expressing Checkpoint Receptor TIGIT Are Enriched in Follicular Lymphoma Tumors and Characterized by Reversible Suppression of T-Cell Receptor Signaling. Clin Cancer Res (2018) 24(4):870–81. doi: 10.1158/1078-0432.CCR-17-2337 PMC5815910 29217528

[132] Judge SJ Darrow MA Thorpe SW Gingrich AA O'Donnell EF Bellini AR. Analysis of Tumor-Infiltrating NK and T Cells Highlights IL-15 Stimulation and TIGIT Blockade as a Combination Immunotherapy Strategy for Soft Tissue Sarcomas. J Immunother Cancer (2020) 8(2):1–17. doi: 10.1136/jitc-2020-001355 PMC7651745 33158916

[133] Kawashima S Inozume T Kawazu M Ueno T Nagasaki J Tanji E. TIGIT/CD155 Axis Mediates Resistance to Immunotherapy in Patients With Melanoma With the Inflamed Tumor Microenvironment. J Immunother Cancer (2021) 9(11):1–13. doi: 10.1136/jitc-2021-003134 PMC8603290 34795004

[134] Liu Z Zhou Q Wang Z Zhang H Zeng H Huang Q. Intratumoral TIGIT(+) CD8(+) T-Cell Infiltration Determines Poor Prognosis and Immune Evasion in Patients With Muscle-Invasive Bladder Cancer. J Immunother Cancer (2020) 8(2):1–9. doi: 10.1136/jitc-2020-000978 PMC7430558 32817209

[135] Raphael I Kumar R McCarl LH Shoger K Wang L Sandlesh P. TIGIT and PD-1 Immune Checkpoint Pathways Are Associated With Patient Outcome and Anti-Tumor Immunity in Glioblastoma. Front Immunol (2021) 12:637146. doi: 10.3389/fimmu.2021.637146 34025646 PMC8137816

[136] Li C Zhao S Zheng Y Han Y Chen X Cheng Z. Preoperative Pembrolizumab Combined With Chemoradiotherapy for Oesophageal Squamous Cell Carcinoma (PALACE-1). Eur J Cancer (2021) 144:232–41. doi: 10.1016/j.ejca.2020.11.039 33373868

[137] Onaitis M. Commentary: Induction Immunotherapy for Esophageal Cancer: A Safe Start. J Thorac Cardiovasc Surg (2021) 161(3):844. doi: 10.1016/j.jtcvs.2020.12.027 33454096

[138] Doki Y Ajani JA Kato K Xu J Wyrwicz L Motoyama S. Nivolumab Combination Therapy in Advanced Esophageal Squamous-Cell Carcinoma. N Engl J Med (2022) 386(5):449–62. doi: 10.1056/NEJMoa2111380 35108470

[139] Tawbi HA Schadendorf D Lipson EJ Ascierto PA Matamala L Castillo Gutiérrez E. Relatlimab and Nivolumab Versus Nivolumab in Untreated Advanced Melanoma. N Engl J Med (2022) 386(1):24–34. doi: 10.1056/NEJMoa2109970 34986285 PMC9844513

[140] Savitsky D Ward R Riordan C Mundt C Wilson NS. Abstract 3819: INCAGN02385 is an Antagonist Antibody Targeting the Co-Inhibitory Receptor LAG-3 for the Treatment of Human Malignancies. Cancer Res (2018) 78(13 Supplement):3819. doi: 10.1158/1538-7445.AM2018-3819

[141] Roudi R D'Angelo A Sirico M Sobhani N. Immunotherapeutic Treatments in Hepatocellular Carcinoma; Achievements, Challenges and Future Prospects. Int Immunopharmacol (2021) 101(Pt A):108322. doi: 10.1016/j.intimp.2021.108322 34735916

[142] Niu J Maurice-Dror C Lee DH Kim DW Nagrial A Voskoboynik M. First-In-Human Phase 1 Study of the Anti-TIGIT Antibody Vibostolimab as Monotherapy or With Pembrolizumab for Advanced Solid Tumors, Including non-Small-Cell Lung Cancer(☆). Ann Oncol (2022) 33(2):169–80. doi: 10.1016/j.annonc.2021.11.002 34800678

[143] Zhao Q Yu J Meng X. A Good Start of Immunotherapy in Esophageal Cancer. Cancer Med (2019) 8(10):4519–26. doi: 10.1002/cam4.2336 PMC6712478 31231980

[144] Yi M Jiao D Xu H Liu Q Zhao W Han X. Biomarkers for Predicting Efficacy of PD-1/PD-L1 Inhibitors. Mol Cancer (2018) 17(1):129. doi: 10.1186/s12943-018-0864-3 30139382 PMC6107958

[145] Doroshow DB Bhalla S Beasley MB Sholl LM Kerr KM Gnjatic S. PD-L1 as a Biomarker of Response to Immune-Checkpoint Inhibitors. Nat Rev Clin Oncol (2021) 18(6):345–62. doi: 10.1038/s41571-021-00473-5 33580222

[146] Shen R Postow MA Adamow M Arora A Hannum M Maher C. LAG-3 Expression on Peripheral Blood Cells Identifies Patients With Poorer Outcomes After Immune Checkpoint Blockade. Sci Transl Med (2021) 13(608):1–12. doi: 10.1126/scitranslmed.abf5107 PMC9254663 34433638

[147] Yang D Han Z Oppenheim JJ. Alarmins and Immunity. Immunol Rev (2017) 280(1):41–56. doi: 10.1111/imr.12577 29027222 PMC5699517

[148] Shang S Liu J Verma V Wu M Welsh J Yu J. Combined Treatment of non-Small Cell Lung Cancer Using Radiotherapy and Immunotherapy: Challenges and Updates. Cancer Commun (Lond) (2021) 41(11):1086–99. doi: 10.1002/cac2.12226 PMC8626591 34658186

[149] Yu WD Sun G Li J Xu J Wang X. Mechanisms and Therapeutic Potentials of Cancer Immunotherapy in Combination With Radiotherapy and/or Chemotherapy. Cancer Lett (2019) 452:66–70. doi: 10.1016/j.canlet.2019.02.048 30902563

